# Supplementary material for: Psychological, pharmacological, and combined treatments for binge eating disorder: a systematic review and meta-analysis
Source: PeerJ. 2018 Jun 21;6:e5113. doi: 10.7717/peerj.5113 (PMC6015752; doi:10.7717/peerj.5113)
Supplement: Supplemental Information 1 — Cochrane Library via Wiley 02 November 2015 (CDSR, DARE & CENTRAL). Updated search on November 2016. Title: Treatment of binge eating disorder. [file peerj-06-5113-s001.docx]

## Appendix A Search strategy

#### Cochrane Library via Wiley 02 November 2015 (CDSR, DARE & CENTRAL)

**Title: Treatment of binge eating disorder**

| Search terms | | Items found |
| --- | --- | --- |
| **Binge eating disorder** | | |
|  | [mh "binge-eating disorder"] | 62 |
|  | binge next eat* or ((binging or bingeing) AND eating):ti,ab,kw | 632 |
|  | 1 OR 2 | 632 |
| **Intervention** | | |
|  | [mh psychotherapy] or [mh counseling] or [mh "directive counseling"] or [mh "treatment outcome"] or [mh "motivational interviewing"] or [mh "treatment failure"] or [mh methylphenidate] OR [mh "rranscranial magnetic stimulation"] OR [mh "fatty acids, omega-3"] OR [mh "nutrition therapy"] OR [mh "nutrition assessment"] or [mh "psychotropic drugs"] or [mh "serotonin uptake inhibitors"] or [mh "self care"] or [mh internet] or [mh "cell phones"] or [mh "text messaging"] | 131429 |
|  | (intervention* or treatment* or therapy or therapies or psychotherapy or behavioral or mindful* or meditation* or "clinical psychology"or "brief intervention" or "brief interventions" or motivation* or medication* or pharmacotherap* or pharmacolog* or antidepressant* or "serotonin uptake" or multimodal OR "web-based” or internet* or phone* or "text messaging" or "text message" or "self-help"or "self-care" or orlistat or fluoxetine or "serotonin uptake inhibitors" or SSRI or topiramate or methylphenidate or "transcranial magnetic stimulation" or "omega-3" or "nutrition therapy" or "nutrition assessment"):ti,ab,kw (Word variations have been searched) | 568927 |
|  | 4 OR 5 | 575003 |
| **Combined sets** | | |
|  | 3 AND 6 = 560 | CDSR/32  DARE/24  Central/495  CRM/2  HTA/4  EED/3 |

The search result, usually found at the end of the documentation, forms the list of abstracts.

[mh] = Mesh

Ti,ab,kw = Title, Abstract, Keywords

* = Truncation

“ “ = Citation Marks; searches for an exact phrase

CDSR = Cochrane Database of Systematic Review

CENTRAL = Cochrane Central Register of Controlled Trials, “trials”

CRM = Method Studies

DARE = Database Abstracts of Reviews of Effects, “other reviews”

EED = Economic Evaluations

HTA = Health Technology Assessments

#### Embase via Elsevier 02 November 2015

**Title: Treatment of binge eating disorder**

| Search terms | | Items found |
| --- | --- | --- |
| **Population: Binge eating disorder** | | |
|  | 'binge eating disorder'/exp/mj | 1929 |
|  | 'binge eating disorder':ab,ti OR 'binge eating syndrome':ab,ti OR (binging NEAR/15 eating):ab,ti OR (bingeing NEAR/15 eating):ab,ti | 2165 |
|  | 1 OR 2 | 3032 |
| **Interventions** | | |
|  | 'drug therapy'/exp/mj OR 'therapy'/exp/mj OR 'counseling'/exp/mj OR 'motivational interviewing'/exp OR 'psychotherapy'/exp/mj OR 'mindfulness'/exp OR 'cognitive therapy'/exp OR 'behavior therapy'/exp OR 'meditation'/exp OR 'psychotropic agent'/exp/mj OR 'drug therapy'/de OR 'anorexigenic agent'/exp OR 'serotonin uptake inhibitor'/exp OR 'transcranial magnetic stimulation'/exp OR 'omega 3 fatty acid'/exp OR 'methylphenidate'/exp OR 'nutritional assessment'/exp OR 'treatment outcome'/exp/mj OR 'treatment failure'/exp/mj OR 'self care'/exp/mj OR 'Internet'/exp OR 'social media'/exp OR 'mobile phone'/exp OR 'text messaging'/exp | 3435684 |
|  | (intervention* OR treatment* OR treating OR therapy OR therapies OR psychotherap* OR mindful* OR behavioral OR behavioural OR meditation* OR clinical psychology OR councel* OR 'brief intervention' OR 'brief interventions' OR motivation* OR medication* OR pharmacotherap* OR pharmacolog* OR antidepressant* OR multimodal OR 'web based' OR internet* OR phone* OR "text messaging" OR "text message" OR "electronic mail" OR 'self help' OR 'self care' OR 'self-directed' OR 'behavioral weight loss' OR orlistat OR fluoxetine OR 'serotonin uptake inhibitors' OR SSRI OR topiramate OR 'transcranial magnetic stimulation' OR 'omega 3' OR 'nutritional assessment' OR methylphenidate OR concerta OR (nutrition* NEXT/2 therap*) OR (nutrition* NEXT/2 intervent*)):ab,ti | 1005551 |
|  | 4 OR 5 | 4032785 |
| **Combined sets** | | |
|  | 3 AND 6 | 1096 |
| **Limits:** | | |
|  | ((animal/exp or nonhuman/exp) NOT human/exp) |  |
|  | [review]/lim |  |
|  | ([article]/lim OR [article in press]/lim) |  |
| **Combined sets** | | |
|  | (7 AND 9) NOT 8 REVIEWS | 242 |
|  | **(7 AND 10) NOT 8** | **642** |

/de= Term from the EMTREE controlled vocabulary

/exp= Includes terms found below this term in the EMTREE hierarchy

/mj = Major Topic

:ab = Abstract

:au = Author

:ti = Article Title

:ti:ab = Title or abstract

* = Truncation

“ “ = Citation Marks; searches for an exact phrase “ “ = Citation Marks; searches for an exact phrase

#### PsycInfo via EBSCO 02 November 2015

**Title: Treatment of binge eating disorder**

| Search terms | | Items found |
| --- | --- | --- |
| **Population: Binge eating disorder** | | |
|  | DE "Binge Eating Disorder" | 1170 |
|  | AB ("binge eating disorder" OR "binge eating syndrome" OR (binging N15 eating) OR (bingeing N15 eating) OR TI ("binge eating disorder" OR "binge eating syndrome" OR (binging N15 eating) OR (bingeing N15 eating) | 2067 |
|  | 1 OR 2 | 2341 |
| **Interventions** | | |
|  | DE "Treatment" OR DE "Adjunctive Treatment" OR DE "Adventure Therapy" OR DE "Aftercare" OR DE "Behavior Modification" OR DE "Cognitive Techniques" OR DE "Computer Assisted Therapy" OR DE "Transcranial Magnetic Stimulation" OR DE "Cross Cultural Treatment" OR DE "Disease Management" OR DE "Health Care Services" OR DE "Interdisciplinary Treatment Approach" OR DE "Life Sustaining Treatment" OR DE "Medical Treatment (General)" OR DE "Milieu Therapy" OR DE "Movement Therapy" OR DE "Multimodal Treatment Approach" OR DE "Multisystemic Therapy" OR DE "Online Therapy" OR DE "Outpatient Treatment" OR DE "Partial Hospitalization" OR DE "Personal Therapy" OR DE "Preventive Medicine" OR DE "Psychotherapeutic Techniques" OR DE "Psychotherapy" OR DE "Rehabilitation" OR DE "Relaxation Therapy" OR DE "Social Casework" OR DE "Sociotherapy" OR DE "Symptoms Based Treatment" OR DE "Treatment Guidelines" OR DE "Self Help Techniques" OR DE "Meditation" OR DE "Mindfulness" OR DE "Self Care Skills OR DE "Drug Therapy" OR DE "Drugs" OR DE "Antidepressant Drugs" OR DE "Serotonin Reuptake Inhibitors" OR DE "Chlorimipramine" OR DE "Citalopram" OR DE "Fluoxetine" OR DE "Fluvoxamine" OR DE "Paroxetine" OR DE "Zimeldine" OR DE "Appetite Depressing Drugs" OR DE "Amphetamine" OR DE "Dextroamphetamine" OR DE "Fenfluramine" OR DE "Phenmetrazine" OR DE "Treatment Outcomes" OR DE "Internet" OR DE "Social Media" OR DE "Online Social Networks" OR DE "Websites" OR DE "Cellular Phones" | 239361 |
|  | AB (intervention* OR treatment* OR treating OR therapy OR therapies OR psychotherapy OR mindful* mindful* OR behavio#ral OR meditation* OR " clinical psychology" OR councel* OR " brief intervention" OR " brief interventions" OR motivation* OR medication* OR pharmacotherap* OR pharmacolog* OR antidepressant* OR orlistat OR fluoxetine OR serotonin uptake inhibitors OR SSRI OR topiramate OR multimodal OR " web based" OR internet* OR " mobile phone" OR " mobile phones" OR "self help" OR " self care" OR 'self-directed' OR 'behavioral weight loss' OR methylphenidate OR concerta OR "transcranial magnetic stimulation" OR OMEGA-3 OR (nutrition* W2 therap*) OR (nutrition* W2 intervent*) OR TI (intervention* OR treatment* OR treating OR therapy OR therapies OR psychotherapy OR mindful* mindful* OR behavio#ral OR meditation* OR " clinical psychology" OR councel* OR " brief intervention" OR " brief interventions" OR motivation* OR medication* OR pharmacotherap* OR pharmacolog* OR antidepressant* OR orlistat OR fluoxetine OR serotonin uptake inhibitors OR SSRI OR topiramate OR multimodal OR " web based" OR internet* OR phone* OR "self help" OR " self care" OR 'self-directed' OR 'behavioral weight loss' OR methylphenidate OR concerta OR "transcranial magnetic stimulation" OR OMEGA-3 OR (nutrition* W2 therap*) OR (nutrition* W2 intervent*)) | 1572535 |
|  | 4 OR 5 | 1604628 |
| **Combined sets** | | |
|  | 3 AND 6 | 1726 |
| **Limits** | | |
|  | **Limiters** - Peer Reviewed; Publication Type: Peer Reviewed Journal; Methodology: LITERATURE REVIEW, -Systematic Review, -Meta Analysis |  |
|  | **Limiters** - Peer Reviewed; Publication Type: Peer Reviewed Journal |  |
|  | 7 AND 8 = Reviews | 125 |
|  | **7 AND 9** | **1292** |

The search result, usually found at the end of the documentation, forms the list of abstracts.

AB = Abstract

AU = Author

DE = Term from the thesaurus

MM = Major Concept

TI = Title

TX = All Text. Performs a keyword search of all the  database's searchable fields

ZC = Methodology Index

* = Truncation

“ “ = Citation Marks; searches for an exact phrase

#### PubMed via NLM 02 November 2015

**Title: Treatment of binge eating disorder**

| Search terms | | Items found |
| --- | --- | --- |
| **Binge eating disorder** | | |
|  | "Binge-Eating Disorder"[Mesh] | 595 |
|  | binge-eating disorder*[Title/Abstract] OR binge eating syndrom*[Title/Abstract] OR ((bingeing[Title/Abstract] OR binging[Title/Abstract]) AND eating[Title/Abstract]) | 2062 |
|  | 1 OR 2 | 2251 |
| **Interventions** | | |
|  | "therapy" [Subheading] OR "Psychotherapy"[Mesh] OR "Counseling"[Mesh:NoExp] OR "Directive Counseling"[Mesh] OR "Motivational Interviewing"[Mesh] OR "Treatment Outcome"[Mesh] OR "Treatment Failure"[Mesh] OR "Psychotropic Drugs"[Mesh] OR "Psychotropic Drugs" [Pharmacological Action] OR "Central Nervous System Stimulants"[Mesh] OR "Serotonin Uptake Inhibitors"[Mesh] OR "Methylphenidate"[Mesh] OR "Transcranial Magnetic Stimulation"[Mesh] OR "Fatty Acids, Omega-3"[Mesh] OR "Nutrition Therapy"[Mesh] OR "Nutrition Assessment"[Mesh] OR Self Care[Mesh] OR "Internet"[Mesh] Or "Social Media"[Mesh] OR "Cell Phones"[Mesh] OR "Electronic Mail"[Mesh] OR "Text Messaging"[Mesh] | 6224647 |
|  | intervention*[Title/Abstract] OR treatment*[Title/Abstract] OR treating[Title/Abstract] OR therapy[Title/Abstract] OR therapies[Title/Abstract] OR psychotherap*[Title/Abstract] OR mindful*[Title/Abstract] OR meditation*[Title/Abstract] OR clinical psychology [Title/Abstract] OR councel*[Title/Abstract] OR brief intervention*[Title/Abstract] OR motivation*[Title/Abstract] OR medication*[Title/Abstract] OR pharmacotherap* [Title/Abstract] OR pharmacolog*[Title/Abstract] OR antidepressant*[Title/Abstract] OR anorexigenic[Title/Abstract] OR multimodal[Title/Abstract] OR web-based[Title/Abstract] OR internet*[Title/Abstract] OR phone*[Title/Abstract] OR text messag*[Title/Abstract] OR self-help[Title/Abstract] OR self-care[Title/Abstract] OR self-directed[Title/Abstract] OR behavioral weight loss[Title/Abstract] OR orlistat[Title/Abstract] OR fluoxetine[Title/Abstract] OR serotonin uptake inhibitors[Title/Abstract] OR SSRI[Title/Abstract] OR topiramate[Title/Abstract] OR methylphenidate[Title/Abstract] OR concerta[Title/Abstract] OR "transcranial magnetic stimulation"[Title/Abstract] OR OMEGA-3[Title/Abstract] OR nutrition therap*[Title/Abstract] OR nutrition intervent*[Title/Abstract] OR nutritional therap*[Title/Abstract] OR nutritional intervent*[Title/Abstract] | 5179081 |
|  | 4 OR 5 | 8567914 |
| **Combined sets** | | |
|  | 3 AND 6 | **1413** |
| **Reviews** | |  |
|  | systematic[sb] | 265203 |
| **Limits** | | |
|  | (animals [MeSH] NOT humans [MeSH]) | 4057397 |
| **Combined sets** | | |
|  | (7 AND 8) NOT 9 = Reviews | 77 |
|  | **7 NOT 9** | **1380** |

The search result, usually found at the end of the documentation, forms the list of abstracts.

[MeSH] = Term from the Medline controlled vocabulary, including terms found below this term in the MeSH hierarchy

[MeSH:NoExp] = Does not include terms found below this term in the MeSH hierarchy

[MAJR] = MeSH Major Topic

[Title/Abstract] = Title or abstract

[TI] = Title

[AU] = Author

[TW] = Text Word

Systematic[SB] = Filter for retrieving systematic reviews

* = Truncation

#### Scopus via Elsevier 02 November 2015

**Title: Treatment of binge eating disorder**

| Search terms | | Items found |
| --- | --- | --- |
| **Binge eating disorder** | | |
|  | TITLE-ABS-KEY  ("binge eating disorder" OR "binge eating syndrome") | 4125 |
| **Interventions** | |  |
|  | TITLE-ABS-KEY (intervention* OR treatment* OR treating OR therapy OR psychotherapy OR counseling OR counselling OR motivational OR "brief intervention" OR "brief interventions" OR multimodal OR antidepressant* OR psychotropic* OR stimulant* OR pharmacolog* OR pharmacotherap* OR SSRI OR "ssri" OR "serotonin uptake inhibitors" OR topiramate OR methylphenidate OR concerta OR "transcranial magnetic stimulation" OR "omega-3" OR "nutrition therapy" OR "nutrition assessment" OR "self care" OR "self help" OR "self directed" OR phone* OR "electronic mail" OR "text messaging" OR "text message" OR "social media" OR internet OR "web-based" OR "behavioral weight loss") | 5654200 |
| **Limits** | |  |
|  | LIMIT-TO ( DOCTYPE ,  **"ar"** )  OR  LIMIT-TO ( DOCTYPE ,  **"re"** ) )  AND  ( LIMIT-TO ( SUBJAREA ,  **"MEDI"** )  OR  LIMIT-TO ( SUBJAREA ,  **"PSYC"** ) ) |  |
|  | **1 AND 2 AND 3** | **2476** |

The search result, usually found at the end of the documentation, forms the list of abstracts.

TITLE-ABS-KEY  = Title or abstract or keywords
ALL = All fields
**PRE/n** "precedes by". The first term in the search must precede the second by a specified number of terms (n).

**W/n** "within". The terms in the search must be within a specified number of terms (n) in any order.
* = Truncation
" " = Citation Marks; searches for an exact phrase
