# Supplementary material for: Psychological, pharmacological, and combined treatments for binge eating disorder: a systematic review and meta-analysis
Source: PeerJ. 2018 Jun 21;6:e5113. doi: 10.7717/peerj.5113 (PMC6015752; doi:10.7717/peerj.5113)
Supplement: Supplemental Information 2 [file peerj-06-5113-s002.docx]

1. Aardoom, J. J., Dingemans, A. E., Spinhoven, P., van Ginkel, J. R., de Rooij, M., & van Furth, E. F. (2016). Web-Based Fully Automated Self-Help With Different Levels of Therapist Support for Individuals With Eating Disorder Symptoms: A Randomized Controlled Trial. *J Med Internet Res, 18*(6), e159.
2. Abiles, V., Rodriguez-Ruiz, S., Abiles, J., Obispo, A., Gandara, N., Luna, V., & Fernandez-Santaella, M. C. (2013). Effectiveness of cognitive-behavioral therapy in morbidity obese candidates for bariatric surgery with and without binge eating disorder. *Nutr Hosp, 28*, 1523-1529.
3. Adriaens, A., Pieters, G., Vancampfort, D., Probst, M., & Vanderlinden, J. (2008). A cognitive-behavioural program (one day a week) for patients with obesity and binge eating disorder: Short-term follow-up data. *Psihologijske Teme, 17*, 361-372.
4. Agh, T., Pawaskar, M., Nagy, B., Lachaine, J., & Voko, Z. (2016). The Cost Effectiveness of Lisdexamfetamine Dimesylate for the Treatment of Binge Eating Disorder in the USA. *Clin Drug Investig, 36*(4), 305-312.
5. Agh, T., Pawaskar, M., Nagy, B., Szilberhorn, L., & Voko, Z. (2015). Budget Impact Analysis of Introducing Lisdexamfetamine Dimesylate for the Treatment of Binge Eating Disorder in the United States. *Value Health, 18*(7), A408.
6. Agras, W. S., Telch, C. F., Arnow, B., Eldredge, K., Detzer, M. J., Henderson, J., & Marnell, M. (1995). Does interpersonal therapy help patients with binge eating disorder who fail to respond to cognitive-behavioral therapy? *Journal of consulting and clinical psychology, 63*, 356-360.
7. Agras, W. S., Telch, C. F., Arnow, B., Eldredge, K., & Marnell, M. (1997). One-year follow-up of cognitive-behavioral therapy for obese individuals with binge eating disorder. *Journal of consulting and clinical psychology, 65*, 343-347.
8. Aguera, Z., Riesco, N., Jimenez-Murcia, S., Islam, M. A., Granero, R., Vicente, E., . . . Fernandez-Aranda, F. (2013). Cognitive behaviour therapy response and dropout rate across purging and nonpurging bulimia nervosa and binge eating disorder: DSM-5 implications. *BMC Psychiatry, 13*, 285.
9. Alger, S. A., Schwalberg, M. D., Bigaouette, J. M., Michalek, A. V., & Howard, L. J. (1991). Effect of a tricyclic antidepressant and opiate antagonist on binge-eating behavior in normoweight bulimic and obese, binge-eating subjects. *American journal of clinical nutrition, 53*, 865-871.
10. Alger-Mayer, S., Rosati, C., Polimeni, J. M., & Malone, M. (2009). Preoperative binge eating status and gastric bypass surgery: A long-term outcome study. *Obes Surg, 19*, 139-145.
11. Allison, K. C., Studt, S. K., Berkowitz, R. I., Hesson, L. A., Moore, R. H., Dubroff, J. G., . . . Stunkard, A. J. (2013). An open-label efficacy trial of escitalopram for night eating syndrome. *Eating Behaviors, 14*, 199-203.
12. Antiepileptic drugs are an emerging option in the management of some eating disorders AUTHOR ADDRESSES SOURCE Drugs and Therapy Perspectives (2009) 25:9 (9-12). Date of Publication: 2009. (2009). *0*.
13. Appolinario, J. C., Bacaltchuk, J., Sichieri, R., Claudino, A. M., Godoy-Matos, A., Morgan, C., . . . Coutinho, W. (2003). A randomized, double-blind, placebo-controlled study of sibutramine in the treatment of binge-eating disorder. *Arch Gen Psychiatry, 60*, 1109-1116.
14. Appolinario, J. C., Coutinho, W., & Fontenelle, L. (2001). Topiramate for binge-eating disorder. *Am J Psychiatry, 158*(6), 967-968.
15. Appolinario, J. C., Fontenelle, L. F., Papelbaum, M., Bueno, J. R., & Coutinho, W. (2002). Topiramate use in obese patients with binge eating disorder: an open study. *Can J Psychiatry, 47*, 271-273.
16. Appolinario, J. C., Godoy-Matos, A., Fontenelle, L. F., Carraro, L., Cabral, M., Vieira, A., & Coutinho, W. (2002). An open-label trial of sibutramine in obese patients with binge-eating disorder. *J Clin Psychiatry, 63*, 28-30.
17. Aragona, M., Pucci, D., & Balbi, A. (2013). [Integrated day-hospital treatment in subjects with overeating disorders]. *Riv Psichiatr, 48*, 315-320.
18. Balestrieri, M., Isola, M., Baiano, M., & Ciano, R. (2013). Psychoeducation in Binge Eating Disorder and EDNOS: a pilot study on the efficacy of a 10-week and a 1-year continuation treatment. *Eat Weight Disord, 18*, 45-51.
19. Banasiak, S. J., Paxton, S. J., & Hay, P. (2005). Guided self-help for bulimia nervosa in primary care: a randomized controlled trial. *Psychological Medicine, 35*, 1283-1294.
20. Barnes, R. D., White, M. A., Martino, S., & Grilo, C. M. (2014). A randomized controlled trial comparing scalable weight loss treatments in primary care. *Obesity (Silver Spring), 22*, 2508-2516.
21. Bauer, C., Fischer, A., & Keller, U. (2006). Effect of sibutramine and of cognitive-behavioural weight loss therapy in obesity and subclinical binge eating disorder. *Diabetes Obes Metab, 8*, 289-295.
22. Birchall, H. (1999). Interpersonal psychotherapy in the treatment of eating disorders. *European eating disorders review, 7*, 315-320.
23. Blom, T. J., Mingione, C. J., Guerdjikova, A. I., Keck, P. E., Jr., Welge, J. A., & McElroy, S. L. (2014). Placebo response in binge eating disorder: a pooled analysis of 10 clinical trials from one research group. *Eur Eat Disord Rev, 22*, 140-146.
24. Blomquist, K. K., & Grilo, C. M. (2011). Predictive significance of changes in dietary restraint in obese patients with binge eating disorder during treatment. *Int J Eat Disord, 44*, 515-523.
25. Boan, J., Kolotkin, R. L., Westman, E. C., McMahon, R. L., & Grant, J. P. (2004). Binge eating, quality of life and physical activity improve after Roux-en-Y gastric bypass for morbid obesity. *Obes Surg, 14*, 341-348.
26. Boutelle, K. N., Zucker, N. L., Peterson, C. B., Rydell, S. A., Cafri, G., & Harnack, L. (2011). Two novel treatments to reduce overeating in overweight children: A randomized controlled trial. *Journal of Consulting and Clinical Psychology S2- Journal of Consulting Psychology, 79*, 759-771.
27. Brauhardt, A., de Zwaan, M., Herpertz, S., Zipfel, S., Svaldi, J., Friederich, H. C., & Hilbert, A. (2015). Therapist adherence in cognitive-behavioral therapy for binge-eating disorder. *Psychotherapeut, 60*(3), 199-204.
28. Brennan, B. P., Roberts, J. L., Fogarty, K. V., Reynolds, K. A., Jonas, J. M., & Hudson, J. I. (2008). Memantine in the treatment of binge eating disorder: an open-label, prospective trial. *Int J Eat Disord, 41*, 520-526.
29. Brownley, K. A., Berkman, N. D., Sedway, J. A., Lohr, K. N., & Bulik, C. M. (2007). Binge eating disorder treatment: a systematic review of randomized controlled trials. *Int J Eat Disord, 40*, 337-348.
30. Busetto, L., Pisent, C., Segato, G., De Marchi, F., Favretti, F., Lise, M., & Enzi, G. (1997). The influence of a new timing strategy of band adjustment on the vomiting frequency and the food consumption of obese women operated with laparoscopic adjustable silicone gastric banding (LAP-BAND). *Obes Surg, 7*, 505-512.
31. Calandra, C., Russo, R. G., & Luca, M. (2012). Bupropion versus sertraline in the treatment of depressive patients with binge eating disorder: retrospective cohort study. *Psychiatr Q, 83*, 177-185.
32. Carter, J. C., & Kelly, A. C. (2015). Autonomous and controlled motivation for eating disorders treatment: Baseline predictors and relationship to treatment outcome. *British Journal of Clinical Psychology S2- British Journal of Social & Clinical Psychology, 54*, 76-90.
33. Castellini, G., Mannucci, E., Lo Sauro, C., Benni, L., Lazzeretti, L., Ravaldi, C., . . . Ricca, V. (2012). Different moderators of cognitive-behavioral therapy on subjective and objective binge eating in bulimia nervosa and binge eating disorder: a three-year follow-up study. *Psychother Psychosom, 81*, 11-20.
34. Castelnuovo, G., Manzoni, G. M., Villa, V., Cesa, G. L., Pietrabissa, G., & Molinari, E. (2011). The STRATOB study: design of a randomized controlled clinical trial of Cognitive Behavioral Therapy and Brief Strategic Therapy with telecare in patients with obesity and binge-eating disorder referred to residential nutritional rehabilitation. *Trials, 12*, 114.
35. Castonguay, L. G., Pincus, A. L., Agras, W. S., & Hines, C. E., III. (1998). The role of emotion in group cognitive-behavioral therapy for binge eating disorder: When things have to feel worse before they get better. *Psychotherapy Research, 8*, 225-238.
36. Cesa, G. L., Manzoni, G. M., Bacchetta, M., Castelnuovo, G., Conti, S., Gaggioli, A., . . . Riva, G. (2013). Virtual reality for enhancing the cognitive behavioral treatment of obesity with binge eating disorder: Randomized controlled study with one-year follow-up. *J Med Internet Res, 15*, 139-151.
37. Chen, E. Y., Matthews, L., Allen, C., Kuo, J. R., & Linehan, M. M. (2008). Dialectical behavior therapy for clients with binge-eating disorder or bulimia nervosa and borderline personality disorder. *Int J Eat Disord, 41*, 505-512.
38. Choudhary, S., & Thapa, K. (2012). Dialectical behavior therapy for managing interpersonal relationships. *Psychological Studies, 57*, 46-54.
39. Compare, A., Callus, E., & Grossi, E. (2012). Mindfulness trait, eating behaviours and body uneasiness: a case-control study of binge eating disorder. *Eat Weight Disord, 17*, e244-251.
40. Crow, S. J. (2003). Group interpersonal psychotherapy may be as effective as group cognitive behavioural therapy for overweight people with binge eating disorder. *Evid Based Ment Health, 6*(2), 56.
41. Dalle Grave, R., Calugi, S., Petroni, M. L., Di Domizio, S., & Marchesini, G. (2010). Weight management, psychological distress and binge eating in obesity. A reappraisal of the problem. *Appetite, 54*, 269-273.
42. Davidsen, A. H., Poulsen, S., Waaddegaard, M., Lindschou, J., & Lau, M. (2014). Feedback versus no feedback in improving patient outcome in group psychotherapy for eating disorders (F-EAT): protocol for a randomized clinical trial. *Trials, 15*, 138.
43. de Zwaan, M., Herpertz, S., Zipfel, S., Tuschen-Caffier, B., Friederich, H. C., Schmidt, F., . . . Hilbert, A. (2012). INTERBED: internet-based guided self-help for overweight and obese patients with full or subsyndromal binge eating disorder. A multicenter randomized controlled trial. *Trials, 13*, 220.
44. DeBar, L. L., Striegel-Moore, R. H., Wilson, G. T., Perrin, N., Yarborough, B. J., Dickerson, J., . . . Kraemer, H. C. (2011). Guided self-help treatment for recurrent binge eating: replication and extension. *Psychiatr Serv, 62*, 367-373.
45. DeBar, L. L., Wilson, G. T., Yarborough, B. J., Burns, B., Oyler, B., Hildebrandt, T., . . . Striegel, R. H. (2013). Cognitive Behavioral Treatment for Recurrent Binge Eating in Adolescent Girls: A Pilot Trial. *Cognitive and behavioral practice, 20*, 147-161.
46. Devlin, M. J., Goldfein, J. A., Carino, J. S., & Wolk, S. L. (2000). Open treatment of overweight binge eaters with phentermine and fluoxetine as an adjunct to cognitive-behavioral therapy. *Int J Eat Disord, 28*, 325-332.
47. Doggrell, S. A. (2008). New indications for topiramate: alcohol dependency and binge-eating disorder. *Expert Opin Pharmacother, 9*, 869-873.
48. Duchesne, M., Appolinario, J. C., Range, B. P., Fandino, J., Moya, T., & Freitas, S. R. (2007). The use of a manual-driven group cognitive behavior therapy in a Brazilian sample of obese individuals with binge-eating disorder. *Rev Bras Psiquiatr, 29*, 23-25.
49. Dunn, E. C., Neighbors, C., & Larimer, M. E. (2006). Motivational enhancement therapy and self-help treatment for binge eaters. *Psychol Addict Behav, 20*, 44-52.
50. Eldredge, K. L., Stewart Agras, W., Arnow, B., Telch, C. F., Bell, S., Castonguay, L., & Marnell, M. (1997). The effects of extending cognitive-behavioral therapy for binge eating disorder among initial treatment nonresponders. *Int J Eat Disord, 21*, 347-352.
51. Fairburn, C. G., Bailey-Straebler, S., Basden, S., Doll, H. A., Jones, R., Murphy, R., . . . Cooper, Z. (2015). A transdiagnostic comparison of enhanced cognitive behaviour therapy (CBT-E) and interpersonal psychotherapy in the treatment of eating disorders. *Behaviour research and therapy, 70*, 64-71.
52. Farci, A. M., Piras, S., Murgia, M., Chessa, A., Restivo, A., Gessa, G. L., & Agabio, R. (2015). Disulfiram for binge eating disorder: an open trail. *Eat Behav, 16*, 84-87.
53. Faulconbridge, L. F., Wadden, T. A., Thomas, J. G., Jones-Corneille, L. R., Sarwer, D. B., & Fabricatore, A. N. (2013). Changes in depression and quality of life in obese individuals with binge eating disorder: bariatric surgery versus lifestyle modification. *Surg Obes Relat Dis, 9*, 790-796.
54. Fichter, M. M., Quadflieg, N., & Gnutzmann, A. (1998). Binge eating disorder: treatment outcome over a 6-year course. *J Psychosom Res, 44*, 385-405.
55. Fichter, M. M., Quadflieg, N., & Hedlund, S. (2008). Long-term course of binge eating disorder and bulimia nervosa: relevance for nosology and diagnostic criteria. *Int J Eat Disord, 41*, 577-586.
56. The following abstracts were presented as posters at the 2014 NEI Psychopharmacology Congress. (2015). *CNS Spectr, 20*, 61-92.
57. Friederich, H. C., Schild, S., Wild, B., de Zwaan, M., Quenter, A., Herzog, W., & Zipfel, S. (2007). Treatment outcome in people with subthreshold compared with full-syndrome binge eating disorder. *Obesity (Silver Spring), 15*, 283-287.
58. Gallagher, M. E., Tasca, G. A., Ritchie, K., Balfour, L., & Bissada, H. (2014). Attachment anxiety moderates the relationship between growth in group cohesion and treatment outcomes in Group Psychodynamic Interpersonal Psychotherapy for women with binge eating disorder. *Group Dynamics: Theory, Research, and Practice, 18*, 38-52.
59. Gelber, D., Levine, J., & Belmaker, R. H. (1999). Inositol treatment on bulimia nervosa and binge eating: a~controlled study. *European neuropsychopharmacology, 9*, S353.
60. Geliebter, A., Hashim, S. A., & Gluck, M. E. (2008). Appetite-related gut peptides, ghrelin, PYY, and GLP-1 in obese women with and without binge eating disorder (BED). *Physiol Behav, 94*, 696-699.
61. Geller, J., Brown, K. E., & Srikameswaran, S. (2011). The efficacy of a brief motivational intervention for individuals with eating disorders: A randomized control trial. *International Journal of Eating Disorders, 44*, 497-505.
62. Ghaderi, A. (2006). Attrition and outcome in self-help treatment for bulimia nervosa and binge eating disorder: a constructive replication. *Eat Behav, 7*, 300-308.
63. Ghaderi, A., & Scott, B. (2003). Pure and guided self-help for full and sub-threshold bulimia nervosa and binge eating disorder. *Br J Clin Psychol, 42*, 257-269.
64. Ginsberg, D. L. (2004). Zonisamide Effective for Binge-Eating Disorder. *Primary Psychiatry, 11*, 14-15.
65. Ginsberg, D. L. (2005). Memantine for binge-eating disorder? Just hold the MSG. *Primary Psychiatry, 12*, 33.
66. Gluck, M. E., Geliebter, A., & Lorence, M. (2004). Cortisol stress response is positively correlated with central obesity in obese women with binge eating disorder (BED) before and after cognitive-behavioral treatment. *Ann N Y Acad Sci, 1032*, 202-207.
67. Goodrick, G. K., Poston, W. S., Kimball, K. T., Reeves, R. S., & Foreyt, J. P. (1998). Nondieting versus dieting treatment for overweight binge-eating women. *Journal of consulting and clinical psychology, 66*, 363-368.
68. Graham, L., & Walton, M. (2011). Investigating the use of CD-Rom CBT for bulimia nervosa and binge eating disorder in an NHS adult outpatient eating disorders service. *Behav Cogn Psychother, 39*, 443-456.
69. Greeno, C. G., & Wing, R. R. (1996). A double-blind, placebo-controlled trial of the effect of fluoxetine on dietary intake in overweight women with and without binge-eating disorder. *Am J Clin Nutr, 64*, 267-273.
70. Grilo, C. (2015). Cardiovascular disease risk reduction in patients with binge eating disorder and obesity: Randomized controlled trial of stepped-care versus standard behavioral weight loss. *Atherosclerosis, 241*(1), e19.
71. Grilo, C. (2016). Randomized controlled trial testing behavioral weight loss versus multi-modal stepped-care treatment for binge eating disorder. *European psychiatry, 33*, S163.
72. Grilo Carlos, M., Crosby Ross, D., Wilson, G. T., & Masheb Robin, M. (2012). 12-month follow-up of fluoxetine and cognitive behavioral therapy for binge eating disorder. [References]. *Journal of consulting and clinical psychology, 80*(6), 1108-1113.
73. Grilo, C. M. (2006). Cognitive behavioural therapy does not improve outcome in obese women with binge eating disorder receiving a comprehensive very low calorie diet programme. *Evid Based Ment Health, 9*(1), 12.
74. Grilo, C. M., Berman, R. M., Daniels, E., McGlashan, T. H., Wilson, G. T., Heninger, G. R., & Masheb, R. (1999). Cognitive-behavior therapy and fluoxetine for binge eating disorder. *152nd Annual Meeting of the American Psychiatric Association. Washington DC, USA. 15-20th May, 1999., 0*.
75. Grilo, C. M., & Masheb, R. M. (2007). Rapid response predicts binge eating and weight loss in binge eating disorder: findings from a controlled trial of orlistat with guided self-help cognitive behavioral therapy. *Behav Res Ther, 45*, 2537-2550.
76. Grilo, C. M., Masheb, R. M., & Crosby, R. D. (2012). Predictors and moderators of response to cognitive behavioral therapy and medication for the treatment of binge eating disorder. *Journal of consulting and clinical psychology, 80*, 897-906.
77. Grilo, C. M., White, M. A., Gueorguieva, R., Wilson, G. T., & Masheb, R. M. (2013). Predictive significance of the overvaluation of shape/weight in obese patients with binge eating disorder: findings from a randomized controlled trial with 12-month follow-up. *Psychol Med, 43*, 1335-1344.
78. Grilo, C. M., White, M. A., Masheb, R. M., & Gueorguieva, R. (2015). Predicting meaningful outcomes to medication and self-help treatments for binge-eating disorder in primary care: The significance of early rapid response. *Journal of consulting and clinical psychology, 83*, 387-394.
79. Grilo, C. M., White, M. A., Wilson, G. T., Gueorguieva, R., & Masheb, R. M. (2012). Rapid response predicts 12-month post-treatment outcomes in binge-eating disorder: theoretical and clinical implications. *Psychol Med, 42*, 807-817.
80. Guerdjikova, A. I., Fitch, A., & McElroy, S. L. (2015). Successful Treatment of Binge Eating Disorder With Combination Phentermine/Topiramate Extended Release. *Prim Care Companion CNS Disord, 17*(2).
81. Guerdjikova, A. I., Kotwal, R., & McElroy, S. L. (2005). Response of recurrent binge eating and weight gain to topiramate in patients with binge eating disorder after bariatric surgery. *Obes Surg, 15*, 273-277.
82. Hilbert, A. (2013). Cognitive-behavioral therapy for binge eating disorder in adolescents: study protocol for a randomized controlled trial. *Trials, 14*, 312.
83. Hilbert, A., Saelens, B. E., Stein, R. I., Mockus, D. S., Welch, R. R., Matt, G. E., & Wilfley, D. E. (2007). Pretreatment and process predictors of outcome in interpersonal and cognitive behavioral psychotherapy for binge eating disorder. *Journal of consulting and clinical psychology, 75*, 645-651.
84. Hill, D. M., Craighead, L. W., & Safer, D. L. (2011). Appetite-focused dialectical behavior therapy for the treatment of binge eating with purging: a preliminary trial. *International Journal of Eating Disorders, 44*, 249-261.
85. Hoopes, S. P., Reimherr, F. W., Hedges, D. W., Rosenthal, N. R., Kamin, M., Karim, R., . . . Karvois, D. (2003). Treatment of Bulimia Nervosa with Topiramate in a Randomized, Double-Blind, Placebo-Controlled Trial, Part 1: Improvement in Binge and Purge Measures. *Journal of Clinical Psychiatry, 64*, 1335-1341.
86. Hudson, J., McElroy, S., Ferreira-Cornwell, C., Radewonuk, J., & Gasior, M. (2015). A double-blind, placebo-controlled, randomized-withdrawal study of lisdexamfetamine dimesylate in adults with moderate to severe binge eating disorder. *Neuropsychopharmacology, 40*, S135-s136.
87. Hugo, P., Segwick, P., Black, A., & Lacey, H. (1999). Telephone counselling - The EDA approach. *European eating disorders review, 7*, 300-309.
88. Jacobi, C., Volker, U., Trockel, M. T., & Taylor, C. B. (2012). Effects of an Internet-based intervention for subthreshold eating disorders: a randomized controlled trial. *Behav Res Ther, 50*, 93-99.
89. Jones, M., Luce, K. H., Osborne, M. I., Taylor, K., Cunning, D., Doyle, A. C., . . . Taylor, C. B. (2008). Randomized, controlled trial of an internet-facilitated intervention for reducing binge eating and overweight in adolescents. *Pediatrics, 121*, 453-462.
90. Keating, L., Tasca, G. A., Gick, M., Ritchie, K., Balfour, L., & Bissada, H. (2014). Change in attachment to the therapy group generalizes to change in individual attachment among women with binge eating disorder. *Psychotherapy (Chic), 51*, 78-87.
91. Klein, A. S., Skinner, J. B., & Hawley, K. M. (2012). Adapted group-based dialectical behaviour therapy for binge eating in a practicing clinic: clinical outcomes and attrition. *Eur Eat Disord Rev, 20*, e148-153.
92. Klein, A. S., Skinner, J. B., & Hawley, K. M. (2013). Targeting binge eating through components of dialectical behavior therapy: preliminary outcomes for individually supported diary card self-monitoring versus group-based DBT. *Psychotherapy (Chic), 50*, 543-552.
93. Kluge, M., Schuld, A., Himmerich, H., Dalal, M., Schacht, A., Wehmeier, P. M., . . . Pollmächer, T. (2007). Clozapine and olanzapine are associated with food craving and binge eating: results from a randomized double-blind study. *Journal of Clinical Psychopharmacology, 27*, 662-666.
94. Kong, S. (2005). Day treatment programme for patients with eating disorders: randomized controlled trial. *J Adv Nurs, 51*, 5-14.
95. Kristeller, J. L., & Hallett, C. B. (1999). An Exploratory Study of a Meditation-based Intervention for Binge Eating Disorder. *J Health Psychol, 4*, 357-363.
96. Lacey, J. H. (1983). Bulimia nervosa, binge eating, and psychogenic vomiting: a controlled treatment study and long term outcome. *British medical journal (Clinical research ed.), 286*, 1609-1613.
97. LaRose, J. G., Fava, J. L., Steeves, E. A., Hecht, J., Wing, R. R., & Raynor, H. A. (2014). Daily self-weighing within a lifestyle intervention: impact on disordered eating symptoms. *Health Psychol, 33*, 297-300.
98. Leombruni, P., Piero, A., Brustolin, A., Mondelli, V., Levi, M., Campisi, S., . . . Fassino, S. (2006). A 12 to 24 weeks pilot study of sertraline treatment in obese women binge eaters. *Hum Psychopharmacol, 21*, 181-188.
99. Lisdexamfetamine for binge eating disorder. (2015). *Current psychiatry, 14*(3), 41-42.
100. Ljotsson, B., Lundin, C., Mitsell, K., Carlbring, P., Ramklint, M., & Ghaderi, A. (2007). Remote treatment of bulimia nervosa and binge eating disorder: a randomized trial of Internet-assisted cognitive behavioural therapy. *Behav Res Ther, 45*, 649-661.
101. Loeb, K. L., Wilson, G. T., Gilbert, J. S., & Labouvie, E. (2000). Guided and unguided self-help for binge eating. *Behav Res Ther, 38*, 259-272.
102. Manasse, S. M., Espel, H. M., Schumacher, L. M., Kerrigan, S. G., Zhang, F., Forman, E. M., & Juarascio, A. S. (2016). Does impulsivity predict outcome in treatment for binge eating disorder? A multimodal investigation. *Appetite, 105*, 172-179.
103. Maranhao, M. F., Estella, N. M., Cury, M. E., Amigo, V. L., Picasso, C. M., Berberian, A., . . . Claudino, A. M. (2015). The effects of repetitive transcranial magnetic stimulation in obese females with binge eating disorder: a protocol for a double-blinded, randomized, sham-controlled trial. *BMC Psychiatry, 15*, 194.
104. Marchesini, G., Natale, S., Chierici, S., Manini, R., Besteghi, L., Di Domizio, S., . . . Melchionda, N. (2002). Effects of cognitive-behavioural therapy on health-related quality of life in obese subjects with and without binge eating disorder. *Int J Obes Relat Metab Disord, 26*, 1261-1267.
105. Marcus, M. D., Wing, R. R., Ewing, L., Kern, E., McDermott, M., & Gooding, W. (1990). A double-blind, placebo-controlled trial of fluoxetine plus behavior modification in the treatment of obese binge-eaters and non-binge-eaters. *American Journal of Psychiatry, 147*, 876-881.
106. Marrazzi, M. A., Markham, K. M., Kinzie, J., & Luby, E. D. (1995). Binge eating disorder: response to naltrexone. *Int J Obes Relat Metab Disord, 19*, 143-145.
107. Masheb, R. M., & Grilo, C. M. (2002). Weight loss expectations in patients with binge-eating disorder. *Obes Res, 10*, 309-314.
108. Masheb, R. M., & Grilo, C. M. (2008). Examination of predictors and moderators for self-help treatments of binge-eating disorder. *Journal of consulting and clinical psychology, 76*, 900-904.
109. Masheb, R. M., Grilo, C. M., & Rolls, B. J. (2011). An RCT for obesity and binge eating disorder: Low-energy-density nutrition counseling and cognitive behavioral therapy. *Obesity (Silver Spring, Md.), 19*, S113-s114.
110. Maxwell, H., Tasca, G. A., Gick, M., Ritchie, K., Balfour, L., & Bissada, H. (2012). The impact of attachment anxiety on interpersonal complementarity in early group therapy interactions among women with binge eating disorder. *Group Dynamics: Theory, Research, and Practice, 16*, 255-271.
111. Maxwell, H., Tasca, G. A., Ritchie, K., Balfour, L., & Bissada, H. (2014). Change in attachment insecurity is related to improved outcomes 1-year post group therapy in women with binge eating disorder. *Psychotherapy (Chic), 51*, 57-65.
112. Mazzeo, S. E., Lydecker, J., Harney, M., Palmberg, A. A., Kelly, N. R., Gow, R. W., . . . edu, s. v. (2016). Development and preliminary effectiveness of an innovative treatment for binge eating in racially diverse adolescent girls. *Eating Behaviors, 22*, 199-205.
113. McElroy, S., Hudson, J. I., Capece, J. A., Beyers, K., Fisher, A. C., & Rosenthal, N. R. (2005). Topiramate for the treatment of moderate to severe binge eating disorder associated with obesity - A double-blind, placebo-controlled study. *Neuropsychopharmacology, 30*, S138.
114. McElroy, S. L., Arnold, L. M., Shapira, N. A., Keck, P. E., Jr., Rosenthal, N. R., Karim, M. R., . . . Hudson, J. I. (2003). Topiramate in the treatment of binge eating disorder associated with obesity: A randomized, placebo-controlled trial: Correction. *The American Journal of Psychiatry S2- American Journal of Insanity, 160*, 612.
115. McElroy, S. L., Guerdjikova, A. I., Winstanley, E. L., O'Melia, A. M., Mori, N., Keck Jr, P. E., & Hudson, J. I. (2011). Sodium oxybate in the treatment of binge eating disorder: An open-label, prospective study. *International Journal of Eating Disorders, 44*, 262-268.
116. McElroy, S. L., Kotwal, R., Hudson, J. I., Nelson, E. B., & Keck Jr, P. E. (2004). Zonisamide in the treatment of binge-eating disorder: An open-label, prospective trial. *Journal of Clinical Psychiatry, 65*, 50-56.
117. McElroy, S. L., Mitchell, J. E., Wilfley, D., Gasior, M., Ferreira-Cornwell, M. C., McKay, M., . . . Hudson, J. I. (2016). Lisdexamfetamine Dimesylate Effects on Binge Eating Behaviour and Obsessive-Compulsive and Impulsive Features in Adults with Binge Eating Disorder. *Eur Eat Disord Rev, 24*(3), 223-231.
118. McElroy, S. L., Mitchell, J. E., Wilfley, D., Gasior, M., Ferreira‐Cornwell, M. C., McKay, M., . . . Hudson, J. I. (2016). Lisdexamfetamine dimesylate effects on binge eating behaviour and obsessive–compulsive and impulsive features in adults with binge eating disorder. *European eating disorders review, 24*(3), 223-231.
119. McElroy, S. L., Shapira, N. A., Arnold, L. M., Keck Jr, P. E., Rosenthal, N. R., Wu, S. C., . . . Hudson, J. I. (2004). Topiramate in the long-term treatment of binge-eating disorder associated with obesity. *Journal of Clinical Psychiatry, 65*, 1463-1469.
120. McElroy, S. L., Shapira, N. A., Arnold, L. M., Keck, P. E., Jr., Rosenthal, N. R., Wu, S.-C., . . . Hudson, J. I. (2005). 'Topiramate in the Long-Term Treatment of Binge-Eating Disorder Associated With Obesity': Correction. *Journal of Clinical Psychiatry S2- Diseases of the Nervous System, 66*, 138.
121. Milano, W., Petrella, C., & Capasso, A. (2005). Treatment of Binge Eating Disorder with sertraline: A randomized controlled trial. *Biomedical research (Tokyo, Japan), 16*, 89-91.
122. Milano, W., Petrella, C., Casella, A., Capasso, A., Carrino, S., & Milano, L. (2005). Use of sibutramine, an inhibitor of the reuptake of serotonin and noradrenaline, in the treatment of binge eating disorder: a placebo-controlled study. *Adv Ther, 22*, 25-31.
123. Mitchell, J. E., Gosnell, B. A., Roerig, J. L., Zwaan, M., Wonderlich, S. A., Crosby, R. D., . . . Wambach, B. N. (2003). Effects of sibutramine on binge eating, hunger, and fullness in a laboratory human feeding paradigm. *Obes Res, 11*, 599-602.
124. Murati, D., Shamsi, H., Knyahnytska, Y., Vincent, M., Abbatangelo, D., Persaud, R., . . . Quilty, L. (2015). Reward sensitivity, impulsivity, and treatment response in binge eating disorder. *Canadian Journal of Diabetes, 39*, S27.
125. Murphy, S., Russell, L., & Waller, G. (2005). Integrated psychodynamic therapy for bulimia nervosa and binge eating disorder: Theory, practice and preliminary findings. *European eating disorders review, 13*, 383-391.
126. Mushquash, A. R., & McMahan, M. (2015). Dialectical behavior therapy skills training reduces binge eating among patients seeking weight-management services: preliminary evidence. *Eating and Weight Disorders, 20*(3), 415-418.
127. Naser, N., McElroy, S., Hudson, J., Mc, F.-C., Radewonuk, J., & Gasior, M. (2015). Lisdexamfetamine dimesylate for adults with moderate to severe binge eating disorder: Results of two randomized controlled safety and efficacy trials. *Australian and New Zealand journal of psychiatry, 49*, 116.
128. Neumeister, A., Winkler, A., & Wober-Bingol, C. (1999). Addition of naltrexone to fluoxetine in the treatment of binge eating disorder. *Am J Psychiatry, 156*(5), 797.
129. Noma, S., Uwatoko, T., Yamamoto, H., & Hayashi, T. (2008). Effects of milnacipran on binge eating--A pilot study. [References]. *Neuropsychiatric disease and treatment, 4*(1), 295-300.
130. O'Reardon, J. P., Allison, K. C., Martino, N. S., Lundgren, J. D., Heo, M., Stunkard, A. J., . . . edu, o. m. m. u. (2006). A randomized, placebo-controlled trial of sertraline in the treatment of night eating syndrome. *American Journal of Psychiatry, 163*, 893-898.
131. O'Reardon, J. P., Groff, K. E., Stunkard, A. J., Allison, K. C., Author, A., Weight, . . . Psychiatry, U. o. P. M. S. P. (2008). Night eating syndrome and results from the first placebo-controlled trial of treatment, with the SSRI medication, sertraline: Implications for clinical practice. *Progress in Neurotherapeutics and Neuropsychopharmacology, 3*, 241-257.
132. Pacanowski, C. R., Senso, M. M., Oriogun, K., Crain, A. L., & Sherwood, N. E. (2014). Binge eating behavior and weight loss maintenance over a 2-year period. *J Obes, 2014*, 249315.
133. Palmer, R. L., Birchall, H., McGrain, L., & Sullivan, V. (2002). Self-help for bulimic disorders: a randomised controlled trial comparing minimal guidance with face-to-face or telephone guidance. *Br J Psychiatry, 181*, 230-235.
134. Park, E. C., Waller, G., & Gannon, K. (2014). Early improvement in eating attitudes during cognitive behavioural therapy for eating disorders: the impact of personality disorder cognitions. *Behav Cogn Psychother, 42*, 224-237.
135. Pataky, Z., Gasteyger, C., Ziegler, O., Rissanen, A., Hanotin, C., & Golay, A. (2013). Efficacy of rimonabant in obese patients with binge eating disorder. *Exp Clin Endocrinol Diabetes, 121*, 20-26.
136. Pendleton, V. R., Goodrick, G. K., Poston, W. S., Reeves, R. S., & Foreyt, J. P. (2002). Exercise augments the effects of cognitive-behavioral therapy in the treatment of binge eating. *Int J Eat Disord, 31*, 172-184.
137. Peterson, C. B., Crosby, R. D., Wonderlich, S. A., Mitchell, J. E., Crow, S. J., & Engel, S. (2013). Predicting group cognitive-behavioral therapy outcome of binge eating disorder using empirical classification. *Behav Res Ther, 51*, 526-532.
138. Pimenta, F., Leal, I., Maroco, J., & Ramos, C. (2012). Brief cognitive-behavioral therapy for weight loss in midlife women: a controlled study with follow-up. *Int J Womens Health, 4*, 559-567.
139. Pisetsky, E. M., Durkin, N. E., Crosby, R. D., Berg, K. C., Mitchell, J. E., Crow, S. J., . . . Peterson, C. B. (2015). Examination of early group dynamics and treatment outcome in a randomized controlled trial of group cognitive behavior therapy for binge eating disorder. *Behav Res Ther, 73*, 74-78.
140. Pla-Sanjuanelo, J., Ferrer-Garcia, M., Gutierrez-Maldonado, J., Riva, G., Andreu-Gracia, A., Dakanalis, A., . . . Sanchez-Planell, L. (2015). Identifying specific cues and contexts related to bingeing behavior for the development of effective virtual environments. *Appetite, 87*, 81-89.
141. Position of the American Dietetic Association: Nutrition intervention in the treatment of anorexia nervosa, bulimia nervosa, and other eating disorders. AUTHOR ADDRESSES SOURCE Journal of the American Dietetic Association (2006) 106:12 (2073-2082). Date of Publication: Dec 2006. (2006). *0*.
142. Raman, J., Hay, P., Tchanturia, K., & Smith, E. (2016). Manualised cognitive remediation therapy for adult obesity: A randomised controlled trial. *Obesity reviews, 17*, 165.
143. Ramanouskaya, T. (2014). Comparison of individual and group cognitive behavioral therapy for binge eating disorder. A randomized, two -year follow-up study. *Obesity facts, 7*, 133.
144. Reas, D. L., & Grilo, C. M. (2008). Review and meta-analysis of pharmacotherapy for binge-eating disorder. *Obesity (Silver Spring), 16*, 2024-2038.
145. Reas, D. L., & Grilo, C. M. (2014). Current and emerging drug treatments for binge eating disorder. *Expert Opin Emerg Drugs, 19*, 99-142.
146. Rigaud, D. J., Brayer, V., Roblot, A., Brindisi, M. C., & Vergès, B. (2011). Efficacy of tube feeding in binge-eating/vomiting patients: a 2-month randomized trial with 1-year follow-up. *JPEN. Journal of parenteral and enteral nutrition, 35*, 356-364.
147. Riva, G., Bacchetta, M., Baruffi, M., Rinaldi, S., Vincelli, F., & Molinari, E. (2000). Virtual reality-based experiential cognitive treatment of obesity and binge-eating disorders. *Clinical Psychology & Psychotherapy, 7*, 209-219.
148. Robinson, A. (2013). Integrative Response Therapy for Binge Eating Disorder. *Cogn Behav Pract, 20*, 93-105.
149. Robinson, P., & Serfaty, M. (2008). Getting better byte by byte: a pilot randomised controlled trial of email therapy for bulimia nervosa and binge eating disorder. *Eur Eat Disord Rev, 16*, 84-93.
150. Roosen, M. A., Safer, D., Adler, S., Cebolla, A., & van Strien, T. (2012). Group dialectical behavior therapy adapted for obese emotional eaters; a pilot study. *Nutr Hosp, 27*, 1141-1147.
151. Rubio, M. A., Author, A., Unidad de Obesidad, N., Clinica Y Dietetica, S. d. E. Y. N. H., Clinico San Carlos, c. M. L. s. n. M. S., Correspondence, A., . . . s/n, M. S. E. m. h. s. m. o. (2009). Topiramate for the long-term treatment of binge eating disorder associated with obesity ORIGINAL (NON-ENGLISH) TITLE Topiramato en el tratamiento a largo plazo del trastorno por atracon asociado a la obesidad. *Revista Espanola de Obesidad, 7*, 402-407.
152. Ruwaard, J., Lange, A., Broeksteeg, J., Renteria-Agirre, A., Schrieken, B., Dolan, C. V., & Emmelkamp, P. (2013). Online cognitive-behavioural treatment of bulimic symptoms: a randomized controlled trial. *Clin Psychol Psychother, 20*, 308-318.
153. Saekow, J., Jones, M., Gibbs, E., Jacobi, C., Fitzsimmons-Craft, E., Wilfley, D., & Barr, T. C. (2015). StudentBodies-eating disorders: A randomized controlled trial of a coached online intervention for subclinical eating disorders. *Internet Interventions, 2*(4), 419-428.
154. Safer, D. L., & Joyce, E. E. (2011). Does rapid response to two group psychotherapies for binge eating disorder predict abstinence? *Behav Res Ther, 49*, 339-345.
155. Safer, D. L., Lively, T. J., Telch, C. F., & Agras, W. S. (2002). Predictors of relapse following successful dialectical behavior therapy for binge eating disorder. *Int J Eat Disord, 32*, 155-163.
156. Schmidt do Prado-Lima, P. A., & Bacaltchuck, J. (2002). Topiramate in treatment-resistant depression and binge-eating disorder. *Bipolar Disord, 4*(4), 271-273.
157. Schmidt, J., & Martin, A. (2016). Neurofeedback against binge eating: A randomized controlled trial in a female subclinical threshold sample. *European eating disorders review, 24*(5), 406-416.
158. Schmidt, U., Lee, S., Beecham, J., Perkins, S., Treasure, J., Yi, I., . . . Eisler, I. (2007). A randomized controlled trial of family therapy and cognitive behavior therapy guided self-care for adolescents with bulimia nervosa and related disorders. *Am J Psychiatry, 164*, 591-598.
159. Shelley-Ummenhofer, J., & MacMillan, P. D. (2007). Cognitive-behavioural treatment for women who binge eat. *Can J Diet Pract Res, 68*, 139-142.
160. Shingleton, R. M., Thompson-Brenner, H., Thompson, D. R., Pratt, E. M., & Franko, D. L. (2015). Gender differences in clinical trials of binge eating disorder: An analysis of aggregated data. *Journal of consulting and clinical psychology, 83*, 382-386.
161. Silveira, R. O., Zanatto, V., Appolinario, J. C., & Kapczinski, F. (2005). An open trial of reboxetine in obese patients with binge eating disorder. *Eat Weight Disord, 10*, e93-96.
162. Smith, E., Raman, J., Tchanturia, K., & Hay, P. (2016). A randomised controlled trial of manualized cognitive remediation therapy in adult obesity. *Obesity facts, 9*, 250.
163. Smith, S. R., Blundell, J. E., Burns, C., Ellero, C., Schroeder, B. E., Kesty, N. C., . . . Weyer, C. (2007). Pramlintide treatment reduces 24-h caloric intake and meal sizes and improves control of eating in obese subjects: a 6-wk translational research study. *American journal of physiology. Endocrinology and metabolism, 293*, E620-627.
164. Sockalingam, S., Cassin, S., Du, C., Wnuk, S., Hawa, R., Jackson, T., & Parikh, S. (2015). A pilot randomized controlled trial of telephone-based cognitive behavioral therapy for preoperative bariatric surgery patients. *Surgery for obesity and related diseases, 11*(6), S53-s54.
165. Steffen, K. J., Mitchell, J. E., le Grange, D., Crow, S. J., Attia, E., Bulik, C. M., . . . Bansal-Dev, V. P. (2010). A prevalence study and description of alli use by patients with eating disorders. *Int J Eat Disord, 43*, 472-479.
166. Steinberg, D. M., Tate, D. F., Bennett, G. G., Ennett, S., Samuel-Hodge, C., & Ward, D. S. (2014). Daily self-weighing and adverse psychological outcomes: a randomized controlled trial. *American journal of preventive medicine, 46*, 24-29.
167. Stice, E., & Ragan, J. (2002). Exercise augments the effects of cognitive-behavioral therapy in the treatment of binge eating. *International Journal of Eating Disorders, 31*, 172-184.
168. Strain, G. W. (2012). Binge eating disorder and bariatric surgery. *J Acad Nutr Diet, 112*(4), 483; author reply 483-484.
169. Striegel-Moore, R. H., Wilson, G. T., DeBar, L., Perrin, N., Lynch, F., Rosselli, F., & Kraemer, H. C. (2010). Cognitive behavioral guided self-help for the treatment of recurrent binge eating. *Journal of consulting and clinical psychology, 78*, 312-321.
170. Stunkard, A. (1994). Binge eating disorder and the treatment of obesity. *Obes Res, 2*(3), 279-280.
171. Stunkard, A., Berkowitz, R., Tanrikut, C., Reiss, E., & Young, L. (1996). d-fenfluramine treatment of binge eating disorder. *Am J Psychiatry, 153*, 1455-1459.
172. Suplicy, H., Boguszewski, C. L., Dos Santos, C. M. C., Do Desterro De Figueiredo, M., Cunha, D. R., & Radominski, R. (2014). A comparative study of five centrally acting drugs on the pharmacological treatment of obesity. *International journal of obesity, 38*, 1097-1103.
173. Sysko, R., Hildebrandt, T., Wilson, G. T., Wilfley, D. E., & Agras, W. S. (2010). Heterogeneity moderates treatment response among patients with binge eating disorder. *Journal of consulting and clinical psychology, 78*, 681-690.
174. Tanofsky-Kraff, M., Shomaker, L. B., Wilfley, D. E., Young, J. F., Sbrocco, T., Stephens, M., . . . Yanovski, J. A. (2014). Targeted prevention of excess weight gain and eating disorders in high-risk adolescent girls: a randomized controlled trial. *Am J Clin Nutr, 100*, 1010-1018.
175. Tasca, G. A., Balfour, L., Ritchie, K., & Bissada, H. (2006). Developmental changes in group climate in two types of group therapy for binge-eating disorder: A growth curve analysis. *Psychotherapy Research, 16*, 499-514.
176. Tasca, G. A., Ritchie, K., Demidenko, N., Balfour, L., Krysanski, V., Weekes, K., . . . Bissada, H. (2013). Matching women with binge eating disorder to group treatment based on attachment anxiety: outcomes and moderating effects. *Psychother Res, 23*(3), 301-314. doi:10.1080/10503307.2012.717309
177. Telch, C. F., Agras, W. S., Rossiter, E. M., Wilfley, D., & Kenardy, J. (1990). Group cognitive-behavioral treatment for the nonpurging bulimic: an initial evaluation. *Journal of consulting and clinical psychology, 58*, 629-635.
178. Tomba, E., Offidani, E., Tecuta, L., Schumann, R., & Ballardini, D. (2014). Psychological well-being in out-patients with eating disorders: a controlled study. *Int J Eat Disord, 47*, 252-258.
179. Traviss, G. D., Heywood-Everett, S., & Hill, A. J. (2011). Guided self-help for disordered eating: A randomised control trial. *Behav Res Ther, 49*, 25-31.
180. Utzinger, L. M., Goldschmidt, A. B., Crosby, R. D., Peterson, C. B., & Wonderlich, S. A. (2016). Are sudden gains important in the treatment of eating disorders? *International Journal of Eating Disorders, 49*(1), 32-35.
181. van Heerden, H. J., Razlog, R., & Pellow, J. (2016). Pilot Study on the Homeopathic Treatment of Binge Eating in Males. *Altern Ther Health Med, 22*, 8-13.
182. Vancampfort, D., De Herdt, A., Vanderlinden, J., Lannoo, M., Soundy, A., Pieters, G., . . . Probst, M. (2014). Health related quality of life, physical fitness and physical activity participation in treatment-seeking obese persons with and without binge eating disorder. *Psychiatry Res, 216*, 97-102.
183. Vancampfort, D., Probst, M., Adriaens, A., Pieters, G., De Hert, M., Stubbs, B., . . . Vanderlinden, J. (2014). Changes in physical activity, physical fitness, self-perception and quality of life following a 6-month physical activity counseling and cognitive behavioral therapy program in outpatients with binge eating disorder. *Psychiatry Res, 219*, 361-366.
184. Vander Wal, J. S., Gang, C. H., Griffing, G. T., & Gadde, K. M. (2012). Escitalopram for treatment of night eating syndrome: A 12-Week, randomized, placebo-controlled trial. *Journal of Clinical Psychopharmacology, 32*, 341-345.
185. Vanderlinden, J., Adriaensen, A., Vancampfort, D., Pieters, G., Probst, M., & Vansteelandt, K. (2012). A cognitive- behavioral therapeutic program for patients with obesity and binge eating disorder: short- and long- term follow-up data of a prospective study. *Behav Modif, 36*, 670-686.
186. Vella-Zarb, R. A., Mills, J. S., Westra, H. A., Carter, J. C., & Keating, L. (2015). A Randomized controlled trial of motivational interviewing + self-help versus psychoeducation + self-help for binge eating. *Int J Eat Disord, 48*, 328-332.
187. Vella‐Zarb, R. A., Mills, J. S., Westra, H. A., Carter, J. C., & Keating, L. (2014). A randomized controlled trial of motivational interviewing + self‐help versus psychoeducation + self‐help for binge eating. *International Journal of Eating Disorders, 0*.
188. Wadden, T. A., Faulconbridge, L. F., Jones-Corneille, L. R., Sarwer, D. B., Fabricatore, A. N., Thomas, J. G., . . . Williams, N. N. (2011). Binge eating disorder and the outcome of bariatric surgery at one year: a prospective, observational study. *Obesity (Silver Spring), 19*, 1220-1228.
189. Wallace, L. M., Masson, P. C., Safer, D. L., & von Ranson, K. M. (2014). Change in emotion regulation during the course of treatment predicts binge abstinence in guided self-help dialectical behavior therapy for binge eating disorder. *J Eat Disord, 2*, 35.
190. Wells, A. M., Garvin, V., Dohm, F. A., & Striegel-Moore, R. H. (1997). Telephone-based guided self-help for binge eating disorder: a feasibility study. *Int J Eat Disord, 21*, 341-346.
191. Wilfley, D. E., Crow, S. J., Hudson, J. I., Mitchell, J. E., Berkowitz, R. I., Blakesley, V., & Walsh, B. T. (2008). Efficacy of sibutramine for the treatment of binge eating disorder: a randomized multicenter placebo-controlled double-blind study. *Am J Psychiatry, 165*, 51-58.
192. Wilson, G. T. (2011). Treatment of binge eating disorder. *Psychiatr Clin North Am, 34*, 773-783.
193. Wilson, G. T., Wilfley, D. E., Agras, W. S., & Bryson, S. W. (2011). Allegiance Bias and Therapist Effects: Results of a Randomized Controlled Trial of Binge Eating Disorder. *Clin Psychol (New York), 18*, 119-125.
194. Wisniewski, L., & Ben-Porath, D. D. (2015). Dialectical behavior therapy and eating disorders: The use of contingency management procedures to manage dialectical dilemmas. *American Journal of Psychotherapy, 69*(2), 129-140.
195. Wnuk, S. M., Greenberg, L., & Dolhanty, J. (2014). Emotion-Focused Group Therapy for Women With Symptoms of Bulimia Nervosa. *Eat Disord, 0*, 1-9.
196. Wolff, G. E., & Clark, M. M. (2001). Changes in eating self-efficacy and body image following cognitive-behavioral group therapy for binge eating disorder: a clinical study. *Eat Behav, 2*, 97-104.
197. Woolhouse, H., Knowles, A., & Crafti, N. (2012). Adding mindfulness to CBT programs for binge eating: a mixed-methods evaluation. *Eat Disord, 20*, 321-339.
198. Ziauddeen, H., Chamberlain, S. R., Nathan, P. J., Koch, A., Maltby, K., Bush, M., . . . Bullmore, E. T. (2013). Effects of the mu-opioid receptor antagonist GSK1521498 on hedonic and consummatory eating behaviour: A proof of mechanism study in binge-eating obese subjects. *Molecular Psychiatry, 18*, 1287-1293.
199. Zunker, C., Peterson, C. B., Cao, L., Mitchell, J. E., Wonderlich, S. A., Crow, S., & Crosby, R. D. (2010). A receiver operator characteristics analysis of treatment outcome in binge eating disorder to identify patterns of rapid response. *Behav Res Ther, 48*, 1227-1231.
