# Supplementary material for: Psychological, pharmacological, and combined treatments for binge eating disorder: a systematic review and meta-analysis
Source: PeerJ. 2018 Jun 21;6:e5113. doi: 10.7717/peerj.5113 (PMC6015752; doi:10.7717/peerj.5113)
Supplement: Supplemental Information 3 — Details of the included studies in terms of population, inclusion criteria, setting, Intervention, duration, dropout, compliance, comparison conditions, duration of treatment, results, and risk of bias including comments on the study. [file peerj-06-5113-s003.docx]

| **First author**  **Year**  **Country**  **Reference** | **Population**  **Inclusion criteria**  **Setting** | **Intervention**  **Duration**  **Drop out**  **Compliance** | **Comparison**  **Duration**  **Drop out**  **Compliance** | **Results**  (at post, if not stated otherwise)  (M±SD, if not stated otherwise) | **Risk of bias**  **Comments**  **Study limitation** |
| --- | --- | --- | --- | --- | --- |
| Agras et al  1994  USA [1]  (1) | **Population,** N=108  (22% of screened)  Mean age: 45±10 (range 22–65)  Age onset of BE: 19  Mean BMI: 38.6±6.6  BE/week: 4.5±1.4  **Inclusion criteria:** Age (not reported), Proposed BED criteria by Walsh (1992), female, no antidepressant medication  **Setting:** Outpatients, via advertisements | CBT+WL, n=36  12 weekly sessions of CBT followed by 18 Weight Loss-sessions  CBT+WL + desipramine (CBT+WL+D), n=36  Desipramine added for the last 6 months, start dos 25 mg/day, max dose 300 mg/day  **Duration:** 36 weeks | WL, n=37  30 group sessions a 90 minutes, weekly for 24 weeks then biweekly  **Duration:** 36 weeks | **Binge days/week**  WL 1.5±0.2, CBT+WL 1.2±1.3,  CBT+WL+D 0.9±0.9  3 months: WL: 2, CBT+WL: 1.7  CBT+WL+D: 1.5  **Remisson**  WL 19%, CBT+WL 37%, CBT+WL+D 41%  3 months: WL 14%, CBT+WL 28%  CBT+WL+D 32%  **Depression** (BDI)  WL 11.3±10.3, CBT+WL: 8.9±7.6  CBT+WL+D, 7.8±7.8  **Weight** (kg)  WL 99.2±16.9, CBT+WL 100±17.6,  CBT+WL+D 105.9±20.5 | **Sampling method**  Unclear  **Blinding**  Assessor blinded  **Handling of missing data**  No ITT |
| Alfonsson et al  2015  Sweden  (2) | **Population,** N=100  (71% of screened)  Mean age: 44±11  Mean BMI: 41±5.3  94% females  **Inclusion criteria:** Age (not reported), Patients with obesity (BMI≥30) and met DSM-5 criteria for BED  **Setting:** Outpatients, routine care at a specialized clinic for obesity | Behavioral Activation, n=50  4–7/group, 10 weekly 90 min sessions following program by Lejuez et al (2010) and Kanter 2009.  **Duration:** 10 weeks | Waiting list, n=50  **Duration:** 3 months  **Drop out:** 12 (24%)  **Compliance:** 38 (76%) | **Remission rates (**zero OBEs past 28 days, based on EDE), n (%)  I 10 (29%), C 10 (26%), ns  **Binge eating,** OBE days  I 7.35±7.03, C 9.29±9.06  3 months: I 4.67±6.17  6 months: I 5.38±7.28  **EDE-Q Total score**  I 3.04±0.84, C 2.95±0.97  **Depression** (HADS)  Post I 5.71±3.83, C 8.05±5.29 | **Sampling method**  Random no list, online randomization  **Blinding**  Blinded assessment  **Handling of missing data**  No ITT analysis |
| Brownley et al  2013  USA  (3) | **Population,** N=24  (58% of screened)  Mean age: 36.6±11  Mean BMI: 43.2±5.4  83.3% females  **Inclusion criteria:** Age 18–65, met DSM-5 criteria for BED, no current suicidal or homicidal intent or other psychiatric conditions, BMI 25–44  **Setting:** Outpatients, recruited via advertisements | Chromium picolinate,  High dose: 1000 µg/day, n=8  Moderate dose, 600 µg/day, n=9  **Duration:** 6 months  **Drop out:** High dose 1,  moderate dose 1 | Placebo, n=7  **Duration:** 6 months  **Drop out:** 1 | **Binge frequency (episodes/months)**  I High dose –1.65±0.76  I Mod dose –0.93±0.70  C –0.97±0.78  **EDE**-**Q Global score**  I High dose –0.21±0.07  I Mod dose –0.13±0.07  C –0.04±0.07  **Depression** (QIDS-SR), decline  I High dose –0.3±0.21  I Mod dose –0.41±0.19  C –0.03±0.21 | **Sampling method**  NR  **Blinding**  Double blind  **Handling of missing data**  NR |
| Carrard et al  2011  Switzerland  (4) | **Population,** N=74  (46% of screened)  Mean age: 36±11.4  BMI: 28.8±5.7  Full BED: 58%  **Inclusion criteria:** Age 18–60, female, average internet skills, met sub-threshold or full DSM-IV criteria for BED, 1 OBE/week for ≥3 months  **Setting:** Outpatients, recruited via advertisements | Internet CBT-gsh, n=37  French programme, based on online programme for BN, SALUT project. Consisted of 11 modules  **Duration:** 6 months  **Drop out:** 9 | Waiting list, n=37  Started treatment after 6 months  **Duration:** 6 months  **Drop out:** 7 | **Abstinence rate n (%)**  I 13(35.1%), C 3(8.1%)  **Objective binge episodes**  I 5.57.4, C 9.1±8.8  **EDE-Q Total**  I 2.5±1.1, C 2.9±1  **Depression** (BDI-II)  I 10±7.4, C 13.2±9.6  **BMI**  I 29.2±6, C 27.9±5.4 | **Sampling method**  Computer generated randomization sequence  **Blinding**  None  **Handling of missing data**  ITT |
| Carter and Fairburn  1998  United Kingdom  (5) | **Population,** N=72  (31% of screened, 95% of eligible)  Mean age: 39.7±10  Mean BMI: 31.4  **Inclusion criteria:** Age 18–65, female, BED according to EDE and research assessment interview  **Setting:** Single centre, outpatient recruited via advertisements | CBT-gsh, n=34  Read and follow the book *Overcoming binge eating.*  Non-specialist therapists as facilitators, between 6–8 25 minute sessions  CBT-sh, n=35  Read and follow the book *Overcoming binge eating*  **Duration:** 12 weeks  **Drop out:** gsh: 8, sh: 0 | Waiting list, n=24  **Duration:** 12 weeks  **Drop out:** 1 | **Binge eating/28 days**  CBT-gsh 4.3±7.8, CBT-sh 9.3±11.7, C 13.5±10.3  3 months: CBT-gsh 3.6±3.5, CBT-sh 5±4.3  6 months: CBT-gsh 3.7±4.2, CBT-sh 4.7±4  **Global EDE-Q score**  CBT-gsh 2.1±1.2, CBT-sh 2.7±1.3, C 3.5±0.8  3 months: CBT-gsh 2.1±1.3, CBT-sh 2.6±1.5  6 months: CBT-gsh 2.4±1.3, CBT-sh 2.6±1.5  **Ceased binge eating, n(%)**  CBT-gsh 17(50%), CBT-sh 15(43%), C 2(8%)  3 months: CBT-gsh 14(41%), CBT-sh 13(37%)  6 months: CBT-gsh 17(50%), CBT-sh 14(40%)  **BMI**  CBT-gsh 31.7±6.1, CBT-sh 30.7±6.6, C 31.9±7.4  3 months: CBT-gsh 30.8±5.9, CBT-sh 29.4±5.6  6 months: CBT-gsh 31.6±6.2, CBT-sh 30.4±6.5 | **Sampling method**  Permuted block by 3  **Blinding**  Assessors blinded  **Handling of missing data**  ITT analysis |
| Cassin et al  2008  Canada  (6) | **Population,** N=108  (84% of eligible)  Mean age: 42.5±12.7  BED duration: 15.1±11.6  Mean BMI: 33.2±7.8  **Inclusion criteria:** Age (NR), Female, met DSM-IV-TR for BED  **Setting:** Outpatients, recruited from local television news, magazine, websites or radio | Adapted Motivational Interviewing (AMI)+SH, n=54  One individual AMI-session + SH book (Treasue and Schmidt 1997) modified for BED  **Duration:** 16 weeks  **Drop out:** 6 (11%) | SH, n=54  Participants were asked to read the book and to complete the worksheets at the initial sessions  **Duration:** 16 weeks  **Drop out:** 8 (15%) | **Binge eating frequency** (days/months)  I 2.8±3.5, C 6.3±6  **Remission, n(%)**  I 15 (27.8%), C 6 (11.1%)  **Depression** (BDI-II)  I 14.2±11.1, C 16.2±12.2 | **Sampling method**  Computerized MINIM program  **Blinding**  Assessor blinded  **Handling of missing data**  ITT analysis |
| Castelnuovo et al  2011  Italy  (7) | **Population,** N=60  (60% of screened)  Mean age: 46±11  Mean weight: 106.95±6.94  100% females  **Inclusion criteria:** Age 18–65, BMI≥30, BED DSM-IV criteria  **Setting:** Consecutive inpatient from single clinical centre | BST, n=30  Inpatient treatment: diet, physical activity, dietician counselling, 8 sessions of BST and 8 outpatient telephone based support sessions  **Duration:** 7 months (1-month inpatient and 6 month outpatient sessions)  **Drop out:** NR | CBT, n=30  Inpatient treatment: diet, physical activity, dietician counselling, 8 sessions of CBT and 8 telephone based outpatient sessions  **Duration:** 7 months (1-month inpatient and 6 months outpatient)  **Drop out:** NR | **BED remission, number of weekly binge episodes <2**  6 months: BST 20%, CBT 63.3%, *p=*.001  **Weight** Change  6 months: CBT –5.95±17.9, BST –10.53±6.14 | **Sampling method**  Web site randomization  **Blinding**  Unclear if assessor is blinded  **Handling of missing data**  Unclear |
| Claudino et al 2007  Brazil  (8) | **Population,** N=73  (91% of eligible)  Mean age: 38  Mean binge days/week: 3.8  Mean BMI: 37.4  35% females  **Inclusion criteria:** Age 18–60, BMI≥30, DSM-IV criteria for BED, >17 BES, not prior been using topiramate  **Setting:** Multicentre, outpatients, spontaneously seeking treatment or recruited via media | Topiramate + group CBT, n=37  *Topiramate*: start dose 25 mg/day for 14 days, increased by 25 mg till target dose of 200 mg/day. Patients presented with <5% weight reduction or <50% reduction in binge episodes were prescribed increased medication, max 300 mg/day.  *CBT*: 19 90-min weekly session (adapted Fairburn model for BED), group of 10, therapist led. Last 3 sessions occurred biweekly  **Duration:** 21 weeks  **Drop out:** 7 (19%) | Placebo + group CBT,  n=36  CBT: 19 90-min weekly session (adapted Fairburn model for BED), group of 10, led by therapist. The last 3 sessions occurred biweekly  **Duration:** 21 weeks  **Drop out:** 10 (20%) | **Binge eating frequency, days/wk**  I 0±0.2, C 0.3±0.6  **Binge episodes/week**  I 0±0.2, C 0.3±0.8  **BED remission**  I 31/37, C 22/36, *p*=.03  **Weight**  Kg: I 89.8±13.2, C 97.5±10.5  BMI: I 35±3.5, C 36.7±4.7, *p*=.0002  **Depression** (BDI)  I 10.9±7, C 9.2±6.9, *p*=0.2  **Adverse events**  Paresthesia (I 48.6%, C 11.1%), Taste perversion (I 24.3%, C 0), Dysuria (I 13.5%, C 0), Insomnia (I 2.7%, C 16.7%) | **Sampling method**  Cluster of ten, computer generated list  **Blinding**  Double blind (topiramate), assessor blinded  **Handling of missing data**  ITT analysis |
| Corwin et al  2012  USA  (9) | **Population,** N=18  Mean age: 44.8±3.7  Mean BMI:37.7±2.3  50% females  **Inclusion criteria:** Adults, self-reported binge eating ≥3 times/week  **Setting:** Outpatients, recruited through website/newsletter | Baclofen, n=12  3 times/day: 10 days to titrate up, 28 days with full dose (20 mg 3 times/day), 10 days to titrate down. Wash out period: 15 days  **Duration:** 48 days  **Drop out:** 5 (28%) | Placebo, n=18  **Duration:** 48 days  **Drop out:** 0 | **Binge frequency** (% days±SEM)  I 0.63±0.08, C 0.78±0.05  **BES score**  I 22.9±3.3, C 21.4±3.1  **Depression** (HAD)  I 7.1±1.2, C 5.8±1  **Adverse events**  Baclofen: 23 reported side effects, Placebo: 11 reported side effects, ns. | **Sampling method**  Unclear  **Blinding**  Double blind  **Handling of missing data**  Unclear |
| Dingemans et al  2007  Netherlands  (10) | **Population,** N=52  Mean age: 37.6  Mean BMI: 38.9±7.9  94% females  **Inclusion criteria:** Age (NR), BED according to the DSM-IV  **Setting:** Outpatients, recruited from 3 eating disorder centres and via advertisements in local newspaper and websites | CBT, n=30  CBT: 15 2 hours’ sessions, the first 10 were weekly, last five were biweekly. Sessions led by two trained therapists  **Duration:** 20 weeks  **Drop out:** 2 (7%) | Waiting list, n=22  Offered CBT after end of treatment  **Duration:** 20 weeks  **Drop out:** 0 | **Objective overeating/28 days**  I 2.1±5.5, C 4.6±6.0  **Subjective BE episodes/28 days**  I 2.3±5.4, C 7.9±13.3  **OBE abstinence, n (%)**  I 19(65%), C 4(18%)  **EDE-Q Global score**  I 1.3±1, C 2.3±0.9  **BMI,** Change  F(1,49)=2.83, *p*=0.1  **Depression** (BDI)  I 12.9±13.2, C 17.4±10.5 | **Sampling method**  Unclear  **Blinding**  Unclear  **Handling of missing data**  NR  **Other Comments**  All analyses were corrected for BL differences between the groups |
| Golay et al  2005  Switzerland  (11) | **Population,** N=89  (91% of screened)  Mean age:41  Mean BMI: 36.6  91% females  **Inclusion criteria:** Age 18–65, BMI≥30, BED met DSM-IV criteria  **Setting:** Outpatients, two centres specializing in obesity and eating disorder, consecutive sample | Orlistat, n=44,  120 mg, TTD with main meal  **Duration:** 24 weeks  **Drop out:** 5(11%) | Placebo, n=45  **Duration:** 24 weeks  **Drop out:** 13(29%) | **BED, % (DSM IV)**  I 9/39 (23%), C 10/34 (29%), *p*=.54  **Binge eating episodes, weekly**  I 1, C 1.7  **Depression** (BDI)  I 8.2±0.8, C 11.6±1.6.2  **QoL** (NHP)  I 5.4±0.8, C 6.8  **Weight loss**  24 week: I –7.4%, C –2.3%, *p*<.0001 | **Sampling method**  Generated by sponsor  **Blinding**  Double blind  **Handling of missing data**  ITT analysis |
| Grilo et al  2014  USA  (12) | **Population,** N=52  Racially ethnically diverse obese individuals  Mean age: 44.5  mean BMI: 37.9  73.4% females  **Inclusion criteria:** Age 18–65, DSM-5 criteria for BED, BMI ≥30–< 50  **Setting:** Primary care, recruited via posters and flyers | CBT-sh + placebo, n=25  *CBT-sh*: primary care physicians instructed participants to read and follow Overcoming Binge eating (Fairburn 1995)  **Duration:** 16 weeks  **Drop out:** Post: 2 (8%), 6 months: 2, 12 months: 4 | Placebo, n=27  **Duration:** 16 weeks  **Drop out:** Post: 7 (26%). 6 moths: 5 (18.5%), 12 months: 4 (15%) | **Remission rates** (zero OBEs past 28 days, EDE interview)  I 6(24%), C 8(29.6%)  6 months: I 10(40%), C 11(40.7%)  12 months: I 11(40%), C 10(37%)  **Binge episodes/months** (EDE)  I 6.4±7.6, C 5.3±9.9  6 months: I 3.6±6.4, C 5.7±10  12 months: I 4.9±8.5, C 6.7±13.9  **EDE-Q Global score**  I 1.71.2, C:2.1±1.2  6 months: I 1.7±0.9, C 1.8±1  12 months: I 1.6±1, C 1.8±1.1  **Depression** (BDI)  I 9.9±9.7, C 9.6±11.3  6 months: I 10.3±10.6, C 8.7±5.1  12 months: I 10.5±9.1, C 7.5±6.9  **BMI**  I 35.9±5.6, C 39.6±5.7  6 months: I 35.3±5.2, C 38.8±5.1  12 months: I 35.4±5.9, C 39.5±5.9 | **Sampling method**  Randomization schedule, stratified by DSM diagnosis of BED  **Blinding**  Unclear  **Handling of missing data**  ITT analysis  **Other comments**  The groups that received sibutramine were excluded since this drug is no longer used in Sweden due to adverse events |
| Grilo et al  2013  USA  (13) | **Population,** N=48  (65% of eligible)  Racially ethnically diverse obese individuals  Full BED, N=34,  Mean age: 45.8±11.0  mean BMI: 37.62±4.79  70% females  **Inclusion criteria:** Age ≤65, DSM-5 criteria for BED, BMI≥30–50  **Setting:** Primary care, recruited via posters and flyers | CBT-sh, n=24  The self-help book *Overcoming Binge eating (Fairburn 1995)*, primary care physicians instructed participants to read and follow the self-help CBT manual  **Duration:** 4 months  **Drop out:** 0 | Usual care, n=24  Advice and treatment recommended by their primary care physicians  **Drop out:** 0 | **Remission rates** (zero OBEs past month, EDE interview)  I 6/24, C 2/24  **Binge frequencies (**EDE, OBE/month)  I 5.75±5.94, C 6.50±7.22  **EDE-Q Global score**  I 3.02±0.88, C 2.81±0.89  **Depression** (BDI)  I 8.88±7.67, C 11.96±7.38  **BMI**  I 37.45±5.34, C 37.42±4.44 | **Sampling method**  Randomization schedule, stratified by DSM diagnosis of BED, blocks of 12  **Blinding**  Unclear  **Handling of missing data**  ITT analysis |
| Grilo et al  2013  USA  (14) | **Population,** N=79  (57% of screened)  Mean age: 46.32±9.68  Mean BMI: 37.57±6.62  82% females  **Inclusion criteria:** Age 21–65, BMI ≥30, BED per DSM5 criteria  **Setting:** Consecutive sample recruited via clinical teams and referrals at a community mental health centre serving economically disadvantaged | Orlistat + BWL, n=20  85% females  *Orlistat*: 120 mg 3 times daily  *BWL*: culturally adaptation of the Diabetes-Prevention-Program, 16 sessions  **Duration:** 4 months  **Drop out:** Post treatment: 1 (5%), 6 months: 2(10%) | Placebo + BWL, n=20  70% females  Placebo: 3 times daily  BWL: culturally adaptation of the Diabetes-Prevention-Program, 16 sessions  **Duration:** 4 months  **Drop out:** Post & 6 months: 1 (5%) | **Remission, n(%)**  I 12(60%), C 14(17%)  6 months: I 10(50%), C 10(50%)  **EDE total**  I 1.6±0.9, C 2.0±0.7  6 months: I 1.5±0.8, C 1.9±1  **BMI**  I 37.9±6.9, C 36±5  6 months: I 37.6±5.7, C 36.7±5.3  **Depression,** (BDI)  I 11.4±12, C 17.7±12  6 months: I 10.3±10.1, C 20.9±11.9 | **Sampling method**  Stratification  **Blinding**  Double blind  **Handling of missing data**  ITT analysis  **Other comments**  Only included data from BED population |
| Grilo et al  2011  USA  (15) | **Population,** N=125  Mean age: 44.8±9.4  Mean BMI: 38.8±5.8  67% females  **Inclusion criteria:** Age 18–60, met full DSM-IV research criteria for BED, BMI 30–55  **Setting:** Outpatients recruited via print advertisements | CBT+ BWL, n=35  CBT was delivered first, followed by BWL.  *CBT*: 16 sessions over 16 weeks, following the protocol by Fairburn 1993.  *BWL*: 16 sessions over 24 weeks following the manualized LEARN programme for weight management (Brownell 2000)  BWL, n=45  16 60-min group sessions for 24 weeks | CBT, n=45  16 group 60-min sessions  **Duration:** 24 weeks  **Drop out:**  Post completion rate: 76% for CBT, 69% for BWL, and 60% for CBT+BWL | **Remission rate** (zero OBEs the last 28 days)  Post: CBT 44.4%, BWL 37.8%, CBT-BWL 48.6%  6-month: CBT 51.1%, BWL 33.3%, CBT-BWL 48.6%  12-month: CBT 51.1%, BWL 35.6%, CBT-BWL 40%  **Binge episodes/months**  Post: CBT 2.2±3.8, BWL 4.6±11, CBT-BWL 3.4±9  6 months: CBT 2.7±8.5, BWL 5.5±7.6, CBT-BWL 3.2±7.8  12 months: CBT 2.4±8.1, BWL 4.6±6, CBT-BWL 4±8.4  **EDE-Q Global score**  Post: CBT 1.7±0.9, BWL 1.8±0.8,  CBT-BWL 1.6±0.9  6 months: CBT 1.6±0.8, BWL 1.8±0.7  CBT-BWL 1.4±0.9  12 months: CBT 1.5±0.8, BWL 1.6±0.8  CBT-BWL 1.4±0.9  **BMI**  Post: CBT 38.5±5.7, BWL 35.7±5.9, CBT-BWL 38.9±6.2  6-month: CBT 38.7±5.7, BWL 36.6±6.8, CBTBWL 38.2±5.3  12-month: CBT 38.3±6, BWL 36.6±6.5, CBT-BWL 38.7±5.6  **Depression (BDI)**  Post: CBT 10.1±8.8, BWL 11.1±8.3, CBT-BWL 9.7±9.2  6 months: CBT 8.1±7.3, BWL 11.1±8.7, CBT-BWL 10.1±9.9  12 months: CBT 9.1±7.9, BWL 9.6±7.7, CBT-BWL 9.7±9.3 | **Sampling method**  No restriction or stratification, computer-generated sequence. Concealed allocation  **Blinding**  Unclear  **Handling of missing data**  ITT analysis |
| Grilo et al  2005  (16)  Masheb et al  2007  (17)  Masheb et al  2008  USA  (18) | **Population,** N=90  Mean age: 46.3±9  Mean BMI: 35.5±7  Age onset BED: 27.1/27.5/28.9  79% females  **Inclusion criteria:** Age 18–60, DSM-IV criteria for BED, BMI≥27  **Setting:** Outpatients recruited via print advertisements | CBT-gsh, n=37  *CBT-gsh*: self-help version of the professional therapist manual (Fairburn, Marcus, & Wilson, 1993)  Behavioural weight loss treatment (BWL-gsh) N=38  *BWL-gsh*: manual is the LEARN Program for Weight Management, 16 lessons  **Duration:** 12 weeks | Placebo, same no of sessions as CBT or BWL, n=15  **Duration:** 12 weeks  **Completers:** 87% | **Remission rate (**No OBEs for past 28 days)  **Self-monitoring, n(%)**  CBT-gsh 17(46%), BWL-gsh 7(18.4%), C 2 (13.3%)  **Remission rate EDE, n(%)**  CBT-gsh: 22(59.5%), BWL-gsh: 9(23.7%), C 4 (26.7%)  **Objective BED/month**  *self-monitored*  CBT-gsh 3.8±6.1, BWL-gsh 7.3±8.2, C 6.8±6.1  *EDE-Q method*  CBT-gsh 2.8±5.1, BWL-gsh 6.7±8, C 8.1±6.9  **Depression (BDI)**  CBT-gsh 9.5±9.4, BWL-gsh 12.0±10.3, C 11.4±8.5  **BMI**  CBT-gsh 33.1±.8, BWL-gsh 34.5±8.8, C 35.8±7.0  **Rapid responder** (≥65% reduction in BE by 4^th^ treatment wk)  CBT-gsh 23/37, BWL-gsh 18/38 | **Sampling method**  5:5:2 ratio, permuted blocks  **Blinding**  Unclear  **Handling of missing data**  ITT analysis |
| Grilo et al  2005  USA  (19) | **Population,** N=50  (29% of screened, 82% of eligible)  Mean age: 47±7 (range 35–58) Mean age onset BED: 25  Mean BMI: 36±4.7  88% females  **Inclusion criteria:** Age 35–60, DSM-IV criteria for BED, BMI≥30  **Setting:** Outpatient, consecutive, via advertisement, Yale University Medical School | Orlistat+CBT-gsh+Diet, n=25  *Orlistat*: 120 mg, 3 times / day  *CBT-gsh:* Using guided self-help and Overcoming Binge Eating (Fairburn 1995). 6 individual meetings (15 – 20 min)  *Diet:* balanced calorie diet women 1200 kcal, men;1500 kcal, fat ≤30%, follow Food Guide Pyramid  **Duration:** 12 weeks  **Drop out:** 6 (24%) | Placebo+CBT-gsh+Diet,  n=25  CBT-gsh and Diet, same as the intervention group  **Duration:** 12 weeks  **Drop out:** 5 (20%) | **Remission rates *(****No OBEs for past 28 days, based on EDE),* *n (%)*  Post: I 16(64%), C (36%), *p* =.048  3 months: I 13(52%), C 13(52%), ns  **Binge Eating, OBEs/Mo**  Post: I 3.2±5.5), C 3.6±5.2), ns  3 months: I 3.4±6.5, C 2.8±5.3), ns  **Depression (BDI)**  Post: I 10.1±7.7, C 14.7±9.0, ns  3 months: I 9.9±8.6, C 14.6±10.9, ns  **EDE Global score**  Post: I 2.1±1, C 2.4±0.7, ns  3 months: I 2.2±1.1, C 2.3±1, ns  **Weight (Kg)**  Post: I –3.5±3.5, C –1.6±2.4, *p* =.02  3 months: I 3.4±5.0, C 1.3±3.1, ns  **Adverse events**  Gastrointestinal events related to Orlistat (flatus with discharge, fatty or oily stools, oily discharge, increased defecation, and faecal urgency) were higher among the Orlistat group than in the placebo group Two patients in the Orlistad+CBT-gsh dropped out due to side effects | **Sampling method**  Computer generated, no restrictions  **Blinding**  Double  **Handling of missing data**  ITT analysis |
| Grilo, Masheb and Wilson  2005  (20)  Grilo et al  2006  (21)  Grilo et al  2012  USA  (22) | **Population,** N=108  (54% of eligible, 26% of screened)  Mean age: 44±8.6 (range 21–59)  Mean BMI: 36.3±7.9  78% females  **Inclusion criteria:** Age 18–60, DSM-IV criteria for BED, 100%–200% of IDW  **Setting:** Consecutive, Outpatient, Yale University, New Haven | Fluoxetine (F), n=27  CBT+placebo (CBT), n=28  CBT+F, n=26  *Fluoxetine:* 60 mg/day (mornings)  *CBT*: weekly individual 60-minutes sessions for 16 weeks, followed Fairburn’s manual for BN  **Duration:** 16 weeks  **Drop out:** 6 (23%)  **Completers:**  F 21(78%),  CBT 20(79%),  CBT+F 20(77%) | Placebo, n=27  **Duration:** 16 weeks  **Drop out:** 5 (19%)  **Completers:** 23 (85%) | **Binge episodes/months (EDE-Q)**  Post: C 7.2±9.2, F 10.3±11.1, CBT 1.8±3.9, CBT+F 4.7±6.9  6 months (EMM±SE): F 11.63±2.37, CBT 5.73±1.43, CBT+F 3.94±1.55  12 months (EMM±SE): F 10.40±1.92, CBT 4.63±1.48, CBT+F 4.64±1.70  **Binge episodes/months (daily self-monitoring)**  Post: C 7.4±10.2, F 11.0±11.2  CBT 2.6±5.8, CBT+F 4.2±6.9  **Remission rate (per EDE)**  Post: C 26%, F 26%, CBT 61%, CBT+F 50%  6 months: F 1/27, CBT 7/28,  CBT+F 9/26,  12 months: F1/27, CBT 10/28, CBT+F 7/26  **EDE-Q Global score**  Post: C 2.1±1.6, F 3.1±1.6, CBT 2.1±1, CBT+F 2.2±1.5  6 months (EMM±SE): F 3.52±0.27, CBT+F 2.5±0.26, CBT 2.5±0.24  12 months (EMM±SE): F 3.32±0.26, CBT+F 2.4±0.25, CBT 2.73±0.24  **Depression (BDI)**  Post: C 11.7±10.3, F 11.8±9.8, CBT 6.5±6.8, CBT+F 9.2±7.3  6 months (EMM±SE): F 14.44±1.67, CBT 10.19±1.49, CBT+F 10.73±1.64  12 months (EMM±SE): F 12.88±1.63, CBT 11.43±1.49, CBT+F 11.17±1.57  **BMI**  Post: C 35.7±7.5, F 38.1±9.6, CBT 34.2±5.8, CBT+F 34.9±7.9  6 months (EMM±SE): F 36.16±0.58, CBT 35.93±0.50, CBT+F 36.86±0.58  12 months (EMM±SE): F 36.15±0.57, CBT 34.76±0.51, CBT+F 35.83±0.58 | **Sampling method**  Computer generated table, blocks of 8, sealed concealment  **Blinding**  Double  **Handling of missing data**  ITT |
| Guerdjikova et al  2012  USA  (23) | **Population,** N=40  (63% of screened)  Mean age: 40±12  Mean BMI: 40.6±7.4  88% females  **Inclusion criteria:** Age 18-65, DSM-IV-TR criteria for BED, ≥2 binge days/week, DSM-IV-TR criteria for depressive disorder: ≥25 on IDS-C  **Setting:** Outpatients, recruited by advertisements, | Duloxetine, n=20  30 mg/day for 7 days, increased to 60 mg/day two weeks. In absence of remission of BE or depressive symptoms, side effects: dose could increase to 90 mg/day forth week and 120 mg/day at beginning of sixth week. Dosing either 1 or 2/day  **Duration:** 12 weeks  **Drop out:** 7 (35%) | Placebo, n=20  Dose same as intervention group  **Duration:** 12 weeks  **Drop out:** 6 (30%) | **Remission**  I 10 (56%), C 6 (30%), ns  **Binge day frequency/week**  I 1.0±1.7 (N=18), C 1.3±1.2 (N=20), *p*=.15  **Binge episode frequency per week**  I 1.1±2.0, C 1.3±1.2, *p*=.27  **BMI**  I 37.7±7.5, C 42.9±7.7, *p*=.08  **Depression**  I 19.1±11.5, C 21.6±12.7, *p*=.32 | **Sampling method**  1:1 ratio, permuted blocks, computer generated  **Blinding**  Double blind  **Handling of missing data**  ITT analysis |
| Guerdjikova et al  2009  USA  (24) | **Population,** N=51  (71% of screened)  Mean age: 44  Mean BMI: 40  79.5% females  **Inclusion criteria:** Age 18–65, met DSM-IV criteria for BED, BMI≥30  **Setting:** Single centre, University Medical centre, Outpatients recruited via advertisements | Lamotrigine, n=26  flexible dose,  84% females  Starting at 25 mg/day for 2 weeks, then successively increased to 100mg/bid if tolerated  **Duration:** 16 weeks (12 weeks dosage titration phase and 4 weeks maintenance phase)  **Drop out:** NR  **Completers**: 14 | Placebo, n=25  75% females  **Duration:** 16 weeks  **Drop out:** NR  **Completers**: 17 | **Remission**  ITT: I 13 (52%), C 18 (75%), *p* =.14  Completers: I 8 (27%), C 16 (94%), *p* =.03  **Binge days/week**  I 1.58±2.12, C 0.76±1.71  **Binges/week**  I 1.65±2.35, C 0.76±1.71  **BMI**  I 38.24±5.7, C 41.50±7.42  **Depression (MADRS)**  I 2.16±3.34, C 0.56±1.23  **EDE-Q Total score**  I 7.96±4.61, C 9.91±4.19  **Adverse events**  No significant difference between the groups. The most common:  Headache: I 35%, C 28%  Insomnia: I 35%, C 20%  Somnolence: I 27%, C 8% | **Sampling method**  1:1 ratio computer generate, permuted blocks  **Blinding**  Double-blind  **Handling of missing data**  ITT analysis |
| Guerdjikova et al  2008  USA  (25) | **Population,** N=44  (79% of screened)  Mean age: 39  Mean BMI: 40.2  98% females  **Inclusion criteria:** Age 18–60, BED according to DSM-IV, BMI≥30  **Setting:** Single centre, outpatients recruited via advertisements | Escitalopram, n=21  10 mg/day for 7 days, then 20 mg/day for 7 days, and then 30 mg/day, remainder of the study. If side effects dosage reduced to 10mg/day  **Duration:** 12 weeks  **Drop out:** 5/20 (25%) | Placebo, n=23  Dose same as for intervention  **Duration:** 12 weeks  **Drop out:** 4 (17%) | **Binges/week**  I 0.9±1.4 (n=20), C 1.7±1.5  **Binge days/week**  I 0.9±1.4, C 1.6±1.4  **Remission**  I 50%, C 26%  **BMI**  I 40.4±7, C 40.5±5  **Depression (HAM-D)**  I 2.4±2.9, C 4.8±5.1  **Adverse events**  No significant difference between the groups. Most common were:  Dry mouth I 33%, C 27%  Diarrhoea I 24%, C 22%  Fatigue I 14%, C 22%  Headache I 14%, C17% | **Sampling method**  1:1 ratio, permuted blocks, computer generated  **Blinding**  Double blind  **Handling of missing data**  ITT analysis |
| Hilbert and Tuschen-Caffier  2004  Germany  (26) | **Population,** N=28  (36% of eligible, 21% of screened)  Full-BED: 71.4%  Mean age: 40  Mean BMI: 35.2  **Inclusion criteria:** Age (NR), DSM-IV criteria for BED except for frequency criterion (freq: 1 day/wk over last 6 months allowed), female  **Setting:** Outpatient recruited via advertisements in newspaper: Psychotherapeutic unit | CBT-E (CBT with body exposure component), n=14  2 hrs sessions, 4–5 members/group. Therapy based on CBT for BN with emphasis on body image disturbance. Sessions conducted by clinical psychologist. Nutritionist and physical therapist also provided services.  **Duration:** 19 weekly session within 5 month and self-management phase of 3 sessions.  **Drop out:** 2 (14.3%)  Duration of BED: 13.5±10.7 years | CBT-C (CBT with cognitive restructuring component focused on body image), n=14  2 hrs sessions, 4–5 members/groups  **Duration:** 19 weekly session within 5 month and self-management phase of 3 sessions  **Drop out:** 2 (14.3%)  Duration of BED: 17.7±13.2 years | **Binges per week past Month**  Post: I 0:6±0:7, C 1:0±1:9  4 months: I 1:2±2:0, C 0:5±1:0  **Binge episodes/28 days, n (%)**  Post: I 0, C 0  4 months: I 0, C 2 (16.6%)  **BED diagnosis, n (%)**  Post: I:2 (16.7%), C 3 (25%)  4 months: I 3 (25%), C 1 (8.3%)  **Remission (abstinent for last 28 days):**  Post: I 4 (33.3%), C 9 (75%)  4 months: I 6 (50%), C 8 (66.7%)  **BMI**  Post: I 33.1±10.4, C 37.2±10.3  4 months: I 33.6±11, C 36.4±11  **Depression (BDI)**  Post: I 12.8±8.8, C 12.7±9.0  4 months I 13.9±8.7, C 12.3±6.9 | **Sampling method**  NR  **Blinding**  unclear if assessor was blinded  **Handling of missing data**  No ITT analysis |
| Hudson et al  1998  USA  (27) | **Population,** N=85  (74% of eligible)  Mean age: 42  Mean BMI: 35  90.5% females  **Inclusion criteria:** Age 18–60, criteria for BED DSM-IV, ≥3 BES/wk for ≥6 months. Number of calories consumed ≥1500 kcal, wt >85% of the midpoint of IDW for Height  **Setting:** Outpatient, 3 centres, recruitment unclear | Fluvoxamine, n=42  50 mg every evening for 3 days. After day 4, dose could be adjusted, individual basis (50–300 mg),  **Duration:** 9 weeks  **Drop out:** 6 | Placebo, n=43  **Duration:** 9 weeks  **Drop out:** 13 | **Remission rate (ITT)**  I 15 (38%), C 11 (26)  **Frequency of binges** (difference between groups, ±SE)  –0.181±0.066, *p*=.006  **Depression** (Hamilton) (difference between groups, ±SE)  –0.401±0.359, *p*=.27  **BMI** (difference between groups, ±SE)  –0.167±0.083, *p*=.04  **Adverse events**  Insomnia: I 44%, C 14%, *p*<.05  Nausea: I 34%, C 12%, *p*<.01  Abnormal dreams: I 20%, C 5% *p*<.01  Headache: I 42%, C 28%  Asthenia: I 32%, C 19% | **Sampling method**  Randomization schedule  **Blinding**  Double  **Handling of missing data**  ITT analysis |
| Kelly et al  2014  Canada  (28) | **Population,** N=41  (69% of eligible)  Mean age 45±15  83% females  **Inclusion criteria:** Age ≥18, DSM-5 criteria for BED, access to internet  **Setting:** Outpatients via advertisements in hospitals and eating disorder community centres, online advertisements | Self-compassion focused therapy (CFT)+food planning, n=15  Behavioural strategies (BS)+food planning, n=13.  Both groups received psychoeducation based on Fairburn’s’ CBT followed by different strategies to cope with their problems  **Duration:** 3 weeks  **Drop out:** CFT:4, BS: 1 | Wait list control group, n=13  **Duration:** 3 weeks  **Drop out:** 1 | **Weekly binge episodes** (±SE)  CFT 2.57±0.84, BS 1.9±0.76, C 4.23±0.78  **Weekly binge days** (±SE)  CFT 1.54±0.51, BS 1.92±0.48, C 3.88±0.49  **EDE–Q Global score**(±SE)  CFT 2.08±0.11, BS 2.45±0.11, C 2.51±0.12  **BMI** (±SE)  CFT 32.67±1.12, BS 33.08±1.11, C 33±1.12  **Depression (CES-D) (±SE)**  CFT 16.64±1.8, BS 19.36±1.75, C 22.92±1.8 | **Sampling method**  Unclear  **Blinding**  Unclear  **Handling of missing data**  ITT analysis |
| Kristeller et al  2014  USA  (29) | **Population,** N=150  (93% of eligible, 70% of screened)  Mean age: 46.6  88% females  Full BED: 66%  **Inclusion criteria:** Age (NR), Full or subthreshold BED, DSM-IV criteria for BED  **Setting**  Two sites, outpatients recruited via advertisements | Mindfulness-based eating awareness training (MB-EAT), n=53  Psycho-educational/cognitive behaviour (PECB), n=50  Both groups had 12 group sessions,1.5 hrs each: 9 weekly and 3 monthly. sessions 1 and 6 were 2 hrs  **Duration:** 9 weeks treatment and then 3 monthly boosters  **Drop out:** MB-EAT 14(26%),  PECB 23(46%) | Waiting list, n=47  **Duration:** same as MB-EAT  **Drop out:** 21 (44%) | **BED diagnosis, ITT**  4 months: MB-EAT 10/31 (32%), PECB 419/35 (54%), C 11/21 (520/31 (64%)  **Abstainers, (not ITT)**  1 month: MB-EAT 9/36 (25%), PECB 10/32 (31%), C 0  4 months: MB-EAT 11/35 (39%), PECB 10/24 (39%), C 5/25 (20%)  **Binge days/months, not ITT**  1 months: MB-EAT 4.78±5.78, PECB 5.23±7.95, C 12.83±8.42  4 months: MB-EAT 3.78±5.15, PECB 5.46±7.67, C 11.38±9.26  **BMI, not ITT**  1 months: MB-EAT 39.54±8.53, PECB 38.95±8.79, C 38.07±6.29  4 months: MB-EAT 40.05±9.21, PECB 38.93±8.99, C 38.42±6.52  **Depression (BDI) not ITT**  1 months: MB-EAT 8.5±9.47, PECB 9.48±10.22, C 17.21±11  4 months: MB-EAT 9.31±11.04, PECB 10±10.37, C 14.12±10.79 | **Sampling method**  Random number  **Blinding**  Unclear  **Handling of missing data**  Mixed models |
| Laederach-Hoffman et al  1999  Switzerland  (30) | **Population,** N=31  (31% of eligible)  Mean age: 38  Mean BMI:39.65  87% females  **Inclusion criteria:** Age 20–60, DSM-IV criteria for BED, obese defined as BMI >27.5  **Setting:** Medical charts of 500 patients were screened, 100 charts were considered suitable, Counselling centre for weight problems | Imipramine, n=15  25 mg 3 times/day for 8 weeks  Thereafter:  *Diet counselling* 30 min individual diet counselling by dietician biweekly.  *Psych Support* –behavioural oriented:  1) individual 15–35 min sessions biweekly  2) group-therapy, 1.5 hours (N=10–14) monthly guided by an assistant dietitian  **Duration:** 8 weeks imipramine, 6 months diet and psych support  **Drop out:** 2 (13%) | Placebo, n=16  for 8 weeks thereafter  *diet counselling and psych support*,  **Duration:** 8 weeks placebo, 6 months diet and psych support  **Drop out:** 1 (6%) | **Binge eating frequency**  Post: I 2.8±3, C 5.4±5.1  6 months: I 4.1±2.1, C 7.2±4.3  **Weight change (kg)**  Post I –2.2±1.8, C 0.2±3.3  6 months: I –1.9±6.3, C 3±2.2  **Depression (HAMD)**  Post: I 9.8±7, C 16±10.3, p<0.001  6 months: I 12.6±5.8, C 19.2±8.7  **Adverse events**:  2 patients | **Sampling method**  NR  **Blinding**  Double  **Handling of missing data**  No ITT analysis  **Funding**  NR |
| Leombruni et al  2008  Italy  (31) | **Population,** N=42  (24% of screened)  Mean age: 39.6±8.5 (range 21–57)  Mean BMI: 39.3±3.5  Duration of BED: 144 months  **Inclusion criteria:** Age 18–65,  Female, BMI ≥30, BED according to DSM-IV-TR, absence of medically unstable conditions, full-syndrome Axis I disorders  **Setting:** Outpatients, recruited from 176 overweight patients from Eating Disorders Pilot Centre, Psychiatric Clinic, University of Turin | Sertraline (S), n=22  25 mg/day for 3 days, then increased in 25-mg increments 3 days to a maximum of 200 mg/day, as tolerated (range 100–200;  All received nutritional training+2 sessions of dietary counselling  **Duration:** 24 weeks  **Drop out:** 6 (27%) | Fluoxetine (F), n=20  10 mg for 3 days, then increased with 10-mg increments every 3 days to a maximum of  80 mg/day (range 40–80 mg).  All received nutritional training+2 sessions of dietary counselling  **Duration:** 24 weeks  **Drop out:** 5 (25%) | **Abstinent**  S 12 (60%), F 9 (52.9%)  **Binge/week**  S 1.1±3.3 (n=16), F 0.9±1.1 (n=15)  **Binge Eating Score ES**  S 15.9±8.2, F 19.2±11.5  **BMI**  S 36.6±4.3, F 38.5±5  **Depression (BDI)**  S 9.9±5.9, F 8.4±6.2  **Side effects (n)**  S 3(15%), F 2(11.8%) | **Sampling method**  Unclear  **Blinding**  Double blind  **Handling of missing data**  No ITT |
| Masheb et al  2011  USA  (32) | **Population,** N=50  Mean age: 45.8±7.6 (range 29–60)  Mean BMI: 39.1±6.6  76% females  **Inclusion criteria:** Age 21–60, met DSM-IV-TR criteria for BED, BMI≥30  **Setting:** Outpatients recruited via advertisements | CBT plus low-Energy-Density diet (CBT+ED), n=25  21 hourly individual sessions (40 minutes devoted to CBT and 20 minutes devoted to energy density): weekly for 1–16 sessions, biweekly thereafter  **Duration:** 6 months | CBT plus General Nutrition counselling (CBT+GN), n=25  21 hourly individual sessions (40 minutes devoted to CBT and 20 minutes devoted to general information about nutrition): weekly for 1–16 sessions, biweekly thereafter  **Duration:** 6 months | **Remission** (0 binges for 28 days prior end of treatment)  *self-monitoring*  CBT+ED 15/25, CBT+GN 18/25  *EDE interview*  CBT+ED 13/25, CBT+GN 11/25  **Binge eating/Months (EDE),** 12 months±SE  CBT+ED 0.7±0.3, CBT+GN 0±0.2  **EDE-Q total score,** ±SE  CBT+ED 1.9±0.2, CBT+GN 2.1±0.2  **Mean change in BMI (ITT)**  6 months: CBT+ED 1.34±2.65, CBT+GN 0.53±1.59  12 months: CBT+ED 1.24±2.65, CBT+GN 0.5±3  **Depression (BDI)** ±SE  CBT+ED 1.9±0.2, CBT+GN 1.8±0.2 | **Sampling method**  Computer-generated randomization schedule concealed allocation  **Blinding**  Unclear if assessor was blinded  **Handling of missing data**  ITT analysis |
| Masson et al  2013  USA  (33) | **Population,** N=60  mean age: 42.8  88.3% females  **Inclusion criteria:** Age ≥18 years, Meet BED criteria or BED criteria with binge eating occurring ≥1week for six months, High school graduate or equivalent, BMI>17.5  **Setting:** Outpatients recruited via advertisements | Guided self-help based on dialectical behaviour therapy (DBT-gsh), n=30  DBT-gsh: received an orientation, DBT manual, and six 20-min support calls over 13 weeks  **Duration:** 13 weeks  **Drop out:** 3/30 | Waiting list, n=30  **Duration:** 13 weeks  **Drop out:** 3/30 | **Objective binge frequencies, last 28 days**  Post: I 5.97 (9.42), C 14.37 (11.86)  **Abstinence, last 28 days**  Post: I 40%, C 3.3%  **EDE-Q Total score, LS**  Post: I 137.30±23.51, C 117.17±17.70 | **Sampling method**  Urn randomization program, stratified based on age and gender  **Blinding**  Assessor was blinded  **Handling of missing data**  ITT analysis |
| McElroy  2015  USA, Sweden, Spain, Germany  (34) | **Population,** N=773  (58% of screened), (study 1: 383, study 2: 390)  Mean age: 38  Mean BMI: 33.5  86% females  **Inclusion criteria:** Age 18–55, moderate to severe BED according to DSM-IV-TR, CGI≥4, BMI ≥18 – ≤45  **Setting:** 2 multicentre, outpatients, via investigator´s databases and local advertisements | Lisdexamfetammine dimesylate (LDX)  Dose optimization for 4 weeks (start dose 30 mg), dose maintenance for 8 weeks (70 mg, could be titrated down to 50 mg if 70 mg was not tolerated)  Study 1, n=192: Study 2, n=195  **Duration:** 12 weeks  **Drop out:** Study 1: 20 (10%)  Study 2: 32 (16%) | Placebo  Study 1, n=191: Study 2, n=195.  **Duration:** 12 weeks  **Drop out:** Study 1: 29/191 (15%), Study 2: 42/192 (21%) | **Binge days/week, change from BL, (**LS mean±SEM)  Study 1: I –3.87±0.124, C –2.51±0.124, *p*<.001  Study 2: I –3.92±0.135, C –2.26±0.137, *p*<.001  **Remisson/cessation**  Study 1: I 76/190 (40%), C 26/184 (14.1%)  Study 2: I 63/174 (36.2%), C 23/176 (13.1%)  **Weight, % change from baseline,** (LS mean±SEM)  Study 1: I –6.25±0.292, C 0.11±0.295  Study 2: I –5.57±0.35, C –1.15±0.353  **Adverse events**  **serious**  Study 1: I 3/192, C 2/187  Study 2: I 1/181, C 2/185 | **Sampling method**  1:1 randomization  **Blinding**  Double blinded  **Handling of missing data**  ITT analysis |
| McElroy  2015  USA  (35) | **Population,** N=60  (43% of screened)  Mean age: 41.3±12  Mean BMI: 40.1±8  Obesity: 92%  85% females  Mean weekly BE: 4.4±1.2  **Inclusion criteria:** Age 18–65, met DSM-IV-TR criteria for BED, 3 BED/week for the last 2 weeks, BMI≥25  **Setting:** Recruited via radio and advertisements | Armodafinil, n=30  flexible dose: Start dose 150mg/day, after 4 weeks if not stopped BE increased to 250 mg/day,  **Duration:** 10 weeks+1 weeks discontinuation  **Drop out:** 8/30 (27%) | Placebo, n=30  **Duration:** 10 weeks  **Drop out:** 3/30/10%) | **Binge days/week, change from BL,**  I –3.1±2.1, C –2.4±1.6  **Depression, IDS, change from BL**  I –5.5±6.9, C –5.5±7.9  **BMI,** **change from BL**  I –0.6±0.8, C 0.1±1.2  **Adverse events**  Headache: I 15/30, C 10/30  Insomnia: I 13/30, C 9/30  Nausea: I 7/30, C 4/30  Felling jittery: I 9/30, C 0  Dry mouth: I 7/30, C 1/30  Anxiety: I 3/30, C 2/30  Fatigue: I 1/30, C 4/30  Diarrhea: I 2/30, C 3/30  Attentions disturbance: I 2/30, C 2/30  Flashes: I 3/30, C 1/30  Vivid dreams: I 2/30, C 1/30  Dizziness: I 1/30, C 2/30  Somnolence: I 2/30, C 1/30 | **Sampling method**  1:1 computer generated coding, permuted blocks, concealed allocation  **Blinding**  Double blinded  **Handling of missing data**  ITT analysis |
| McElroy et al  2015  USA  (36) | **Population,** N=260  (42% of screened, 83% of eligible)  Mean age 38.7±10.2  Mean BMI.34.9±5.3  81.5% females  **Inclusion criteria:** Age 18–55, met DSM-IV TR criteria for BED, BMI≥25–45  **Setting:** 31 sites, outpatients, recruitment unclear | Lisdexamfetamine  30, 50 or 70 mg/day  Dosage titrated across 3 weeks and maintained for 8 weeks  30 mg/day: n=66  50 mg/day, n=65  70 mg/day, n=65  **Duration:** Study duration 14 weeks, Treatment 11 weeks  **Drop out:**  30 mg/day: N=15 (23%)  50 mg/day, N=13 (20%)  70 mg/day, N=13 (20%) | Placebo, n=63  **Duration:** 14 weeks  **Drop out:** 17 (27%) | **BE days/week**  Placebo 1.1±1.45, 30 mg/day 1±1.69,  50 mg/day 0.4±0.86, 70 mg/day 0.5±1.25  **Change**  Placebo –3.3±2.04, 30 mg/day –3.5±1.95,  50 mg/day –4.1±1.52, 70 mg/day: 4.1±1.57  **BE episodes**  Placebo 1.1±1.55, 30 mg/day 1.2±2.13,  50 mg/day 0.5±1.01, 70 mg/day0.5±1.34  **Remission**  Placebo 21.3%, 30 mg/day 34.9%,  50 mg/day 42.4%, 70 mg/day 50%  **Depression (MADRS)** (change±SE)  Placebo –1.7±0.35, 30 mg/day –1.9±0.34,  50 mg/day –1.3±0.33, 70 mg/day –1.6±0.33  **Weight, kg mean change**  Placebo –0.1±3.09, 30 mg/day –3.1±3.64,  50 mg/day –4.9±4.43, 70 mg/day –4.9±3.93  **Adverse events**  Any: Placebo 58%, 30 mg/day 86.4%,  50 mg/day 86.2%, 70 mg/day 81.5%,  Dry mouth: Placebo 7.9%, 30 mg/day 33.3%, 50 mg/day 33.8%, 70 mg/day 41.5%  Decreased appetite: Placebo 6.3%, 30 mg/day 25.8%, 50 mg/day 20%, 70 mg/day 18.5%  Insomnia: Placebo 1.6%, 30 mg/day 10.6%, 50 mg/day 15.4%, 70 mg/day 13.8% | **Sampling method**  1:1:1, web response system  **Blinding**  Double blind  **Handling of missing data**  ITT analysis |
| McElroy et al  2007  USA  (37) | **Population,** N=394  Mean age: 44.5  Mean BMI: 38.5  84.2% females  **Inclusion criteria:** Age 18–65 years, met DSM-IV criteria for BED, ≥3 BE days/week, BMI 30–50  **Setting:** Single centre, outpatients from private practice and university, recruited via advertisements and radio | Topiramate, n=195  Flexible dose; 25 mg/day, titrated weekly over an 8-week period to 400 mg/day or the max tolerated dose.  **Duration:** 16 weeks  **Drop out:** 55 (27%) | Placebo, n=199  **Duration:** 16 weeks  **Drop out:** 59 (29%) | **Remission**  I 113 (58%), C 57 (29%)  **Binge eating days/week**  Mean change: I –3.5±1.9, C –2.5±2.1, *p*<.001  I 0.9, C 2.2  **Depression (MADRS)**  Mean change: I –0±7, C –0.7±6.2  **BMI**  I -1.6±1.8, C 1 ±1.2, p<0.01  **Adverse events** (*p*<.001)  Parasthesia: I 55.9%, C 12.4%  Taste perversion: I 13.9%, C 1,0% Concentration problems: I 12.9%, C 2.5% | **Sampling method**  1:1 ratio, permuted block, computer generated  **Blinding**  Double blind  **Handling of missing data**  ITT analysis |
| McElroy, Hudson et  Al  2003  USA  (38) | **Population,** N=38  (76% of screened)  Lifetime major depressive disorder: 86.5%  Current major depressive  disorder: 31%  95% Females  **Inclusion criteria:** Age 18–60, DSM-IV criteria for BED, ≥3 binge episodes weekly for ≥6 months: weight >85% of IBW  **Setting:** Outpatients recruited via advertisement, single center | Citalopram, n=19  20 mg/day for first 7 days: increased as tolerated to 40 mg/day for 7 days, and then 60 mg/day for remainder of study.  Mean age: 42.0±9.0  **Duration:** 6 weeks  **Drop out:** 3 (16%) | Placebo, n=19  Mean age:39.2±12.0  95% females  **Duration:** 6 weeks  **Drop out:** 4 (22%) | **Binges/week**  I 1.7±3.1, C 3.4±3.0  **Binge days/week**  I 1.2±2.0, C 2.8±2.2  Remission  I 9/19 (47%), C 2/19 (21%)  **Depression (HAM-D)**  I 1.4±2.3, C 1.9±3.1  **BMI**  I 40.9±7.0, C 35.7±7.5  **Adverse events:** Sweating (*p*=.008), fatigue (*p* =.046), dry mouth, headache, diarrhoea, nausea, sedation, insomnia, sexual dysfunction | **Sampling method**  Unclear  **Blinding**  Double  **Handling of missing data**  ITT analysis |
| McElroy et al  2000  USA  (39) | **Population,** N=34  Mean age: 42  Mean BMI: 36.1  94.5% females  **Inclusion criteria:** Age 18–60, DSM-IV criteria for BED, ≥3 BE/week for ≥6 months, BE defined by DSM-IV criteria plus required size at least 1500 kcal weight >85% of IBW  **Setting:** Outpatient, single centre, recruitment unclear | Sertraline, n=18  50mg/day for ≥3 days: adjusted as tolerated to between 1 to 4 capsules daily. Mean end of study  dose 187±30 mg  89% females  **Duration:** 6 weeks  **Drop out:** 5 (28%) | Placebo, n=16  100% females  **Duration:** 6 weeks  **Drop out:** 3 (19%) | **Binge/week**  Post: I 1.13±1.56, C 3.85±3.81, *p*=.008  **Frequency of binges**  Remission/cessation of binges: I 7, C 2  **BMI**  Diff between groups in change  over time (SE): –0.596 (0.189), *p*=.002 in favour of I  **Depression (HAMD)**  Diff between groups over time  (SE): 1.33 (1.00)  **Advert Events:**  Insomnia, I 7 (39%), C 1 (6%), *p*=.04 | **Sampling method**  Unclear  **Blinding**  Double  **Handling of missing data**  ITT analysis |
| McIntosh 2016  USA New Zealand  (40) | **Population,** N=112  Mean age: 35.2±12.6  Mean BMI: 2.9±7.8  100% females  **Inclusion criteria:** Age 16–65, Females, primary DSM-IV episodes  **Setting:** Recruited by referrals from general practitioners or other health professionals and by advertisements | **Treatment 1** CBT, n=38  Divided in 3 overlapping phases. Phase 1 introduction to CBT, Phase 2; further CBT skills. Written psychoeducational materials are provided. Phase 3; information on the relapse and recovery process, teaching strategies to reduce risk of relapse  **Drop out:** 6 months n=5, 12 months n=6  **Treatment 2** Appetite-focused CBT (CBT-A), n=36  CBT + strategies to recognize and respond to hunger and satiety cues in the return to normal eating  **Drop out:** 6 months n=6, 12 months n=4  **Duration:** 12 months; six months of weekly individual psychotherapy sessions, followed by six months of monthly sessions | Schema therapy (ST),  n=38  Modifying maladaptive schemas to enable core psychological needs to be met and to bring change in the eating disorder.  **Duration:** Six months of weekly individual psychotherapy sessions, followed by six months of monthly sessions  **Drop out:** 6 months n=9, 12 months n=4 | **Binge frequency**  Post: CBT 7.3±13.9, ST 5±13.2, CBT-A 6.5±13, ns  12 months: CBT 7.3±18.9, ST 4.3±10.4, CBT-A 3.6±9.5, ns  **EDE Global**  Post: CBT 1.7±1.3, ST 2±1.3, CBT-A 1.9±1.7, ns  12 months: CBT 1.3±1.3, ST 1.6±1, CBT-A 1.4±1.2, ns  **BMI**  Post: CBT 30.5±8.6, ST 30.5±8.2, CBT-A 29.5±7.3  12 months: CBT 30.3±8.3, ST 30.4±7.7, CBT-A 30.4±7.6, ns  **GAF**  Post: CBT 67.2±14.2, ST 71.2±12.5, CBT-A 69.9±16.3, ns  12 months: CBT 72.2±16.4, ST 71.5±14.8, CBT-A 70.5±16.1 | **Sampling method**  1:1:1 ratio based on a randomization sequence of permuted blocks of 30.  Sealed envelopes  **Blinding**  Unblinded |
| Munsch et al  2007  (41)  Munsch et al  2012  Switzerland  (42) | **Population,** N=80  (80% of eligible, 21 of screened)  Mean age: 46.1  Mean BMI: 34  89% females  **Inclusion criteria:** Age 18–70, BMI 27–40, meet full DSM-IV-TR1 criteria for BED  **Setting:** Outpatients recruited via newspaper advertisements | CBT, n=44  For both CBT and BWL:  ≤7 members/group, therapist led, active treatment phase: 16 weekly 90-min sessions. Follow-up treatment: 6 monthly 90-min sessions. Final session 12 months after the end of active treatment.  **Duration:** Active: 16 weeks  **Drop out:** 16 weeks: 13 (29.3%). FU 12 months: 15 (34%). 6 years: 23 (52%) | BWL, n=36  **Duration:** 16 weeks  **Drop out:** 16 weeks: 9 (25.0%). FU 12 months: 13 (36%). 6 years: 18 (50%) | **Objective binge days/28 days**  Post: CBT 6.20±8.66, BWL 7.54±9.38  12 months: CBT 4.8±4 8, BWL 5.77±9.15  **BED diagnosis, %**  Post: CBT 50%, BWL 78%  12 months: CBT 43%, BWL 53%  6 years: CBT 3.8%(1/26), BWL 11.5% (3/2)  **Remission**  Post: CBT 41%, BWL 58%  12 months: CBT 52%, BWL 50%  **No of weekly binges (self-reported)**  Post: CBT 0.14±0.45, BWL 1.15±1.89  12 months: CBT 0.52±1.59, BWL 1.5±2.14  **BMI**  Post: CBT 33.58±4.53, BWL 32.±29 4  12 months: CBT 33.1±5.04, BWL 33.18±4.17  6 years: CBT 31.5±5.2, BWL 33.5±3.8  **Depression (BDI)**  Post: CBT 9.16±7.8, BWL 9.19±6.54  12 months: CBT 8.23±11.31, BWL 7.76±6.48 | **Sampling method**  Permuted block design  **Blinding**  Assessors blinded  **Handling of missing data**  ITT analysis |
| Pearlstein et al  2003  USA  (43) | **Population,** N=20  (80% of screened)  Mean age: 41.0  Mean BMI: 41.16  85% females  **Inclusion criteria:** Age (NR), DSM IV research criteria for BED based on EDE  **Setting**: Outpatient recruited via advertisements and referral from health professional, single centre | Fluvoxamine, n=9  flexible dose, titrated up to 150 mg bid.  Average dose 239 mg/day,  **Duration:** 12 weeks  **Drop out:** 0 | Placebo, n=11  Average dose 264 mg/day  **Duration:** 12 weeks  **Drop out:** 0 | **Remission**  I 50%, C 50%  **Binge days/past 28 days**  I 3.11±4.20, C 7.31±9.31  **Depression (HAM-D)**  I 9.38±9.71, C 7.38±9.71  **Weight, lbs**  I 242±82, C 262±99  **Adverse events, N:**  In study completers,  Sedation: I 8, C 3  Nausea: I 4, C 1  Dry mouth: I 4, C 3  Decreased libido: I 3, C 0 | **Sampling method**  Unclear  **Blinding**  Double  **Handling of missing data**  Unclear |
| Peterson et al  2009  USA  (44) | **Population,** N=129  Mean age: 48  Mean BMI: 38.7  90% females  Antidepressant medication: 78.8%  **Inclusion criteria:** Adults, DSM-IV full criteria for BED, BMI≥25  **Setting:** Recruited from two Midwestern clinical sites using advertisements and referrals from local eating disorder treatment clinics and other health professionals | Therapist-led CBT, n=60  15 group sessions of 80-minute duration over a 20-week period, weekly sessions for the first 10 weeks and then bi-weekly group size: 2–11  **Duration:** 20 week  **Drop out:** 7/60 (12%) | Waiting list, n=69  **Duration:** 20 weeks thereafter started treatment  **Drop out:** 13/69 (19%) | **Abstinence/remission rate**  Post: C 10%, I 52%  6 months: I 43%, 12 months: I 21%  **OBE days/28 days**  Post: C 13.5±9.3, I 4.4±7.3  6 months: I 7.4±9.3, 12 months: I 10.6±9.3  **OBE episodes**  Post: C 17.6±14.6, I 6.3±12.3  6 months: I 10.6±14.8,  12 months: I 16.2±19.4  **EDE-Q Global score**  Post: I 2.1±0.9, C 2.3±0.9  6 months: I 2.1±0.9,  12 months: I 2.4±1  **BMI**  Post: C 38.3±7.4, I 40.8±11.7  6 months: I 39.8±10.0  12 months: I 38.3±8.5  **Depression (IDS-SR 22)**  Post: C: 23.3±10.7, I: 19.8±11.3  6 months: I: 20.3±11.7  12 months: I: 20.8±12 | **Sampling method**  By independent bio-statistician, adaptive randomization strategy, concealed  **Blinding**  Assessors blinded  **Handling of missing data**  Unclear  **Other comments**  We only included the group therapist-led condition and waitlist since the other groups had too high drop out to be included. |
| Ricca et al  2010  Italy  (45) | **Population,** N=144  (91% of eligible, 82% of screened)  Mean age: 47  88.2% females  Sub-BED: 43.8%  **Inclusion criteria:** Age 18–60, meet DSM-IV criteria for BED or subthreshold BED  **Setting:** Outpatients, Clinic for Eating Disorders, recruited via referrals by family doctors and other clinicians | Individual CBT, n=72  22 individual sessions of 50 min each, group size:12  **Duration:** 24 weeks  **Drop out:** 4/72 (BED patients) | Group CBT (G-CBT), n=72  20 group sessions of 60 min each, group size:12  **Duration:** 22 weeks  **Drop out:** 6/72 | **Remission rate**  Post: CBT 33.3%, G-CBT 16.7%  3 years: CBT 36%, G-CBT 28%  **Diagnostic change from BED to s-BED**  CBT 18.1%, G-CBT 33.3%  **Binge episodes/months,** Md (quartiles)  Post: CBT 4.0 (0;7.5), G-CBT 4.0 (2.0;8.0)  3 years: CBT 4.0 (0;6), G-CBT 4.0 (0;8)  **BMI,** mean  Post: CBT 36.5 (32.1;42.3)  G-CBT 37.4 (32;40.1)  3 years: CBT 36.0 (3;42.7), G-CBT 37 (31.9;41.8)  **Depression (BDI)** Md (quartiles)  Post: CBT 17 (12;23), G-CBT 15, (9;22.7)  3 years: CBT 17 (11.7;1.5), G-CBT 14.0 (7;22)  **EDE-Q Total,** Md (quartiles)  Post: CBT 2.1 (0.5;3.3), G-CBT 2.9 (2.3;3.5)  3 years: CBT 1.3 (0.5;3.1), G-CBT 2.7 (2.1;3.4)  **QoL (SCL-90)** Md (quartiles**)**  Post: CBT 1.2 (0.7;1.7)  G-CBT 1.1 (0.7;1.3)  3 years: CBT 1.2 (0.6;1.7)  G-CBT 1.1 (0.7;1.3)  **BES,** Md (quartiles)  Post: CBT 16 (3.5;30), G-CBT 17.0 (12;27.7)  3 years: CBT 17.5 (12.0;31), G-CBT 17 (11;25) | **Sampling method**  Table of random no, permuted blocks  **Blinding**  The assessors were blinded  **Handling of missing data**  ITT analysis |
| Schlup et al  2009  (46)  Fischer et al  2014  Switzerland  (47) | **Population,** N=36  (78% of eligible, 27% of screened)  Mean age: 44.3±10.3  Mean BMI: 33.4±7.6  **Inclusion criteria:** Age 18–70, met DSM-IV-TR full diagnostic criteria for BED  **Setting:** Recruited via newspaper advertisements and flyers, University of Basel | Short version of CBT (group) + booster sessions, n=18  8 weekly 90-min sessions  **Duration:** 8 weeks  **Drop out:** 1/18 | Waiting list, n=18  **Duration:** 8 weeks  **Drop out:** 0 | **Abstainer rates, %**  CBT 39%, C 0%  **Objective binge episodes (difference from BL)**  CBT –5.47, C –0.43  **Subjective binge episodes, (difference from BL)**  CBT–0.65, C –0.17  **No of weekly binges (difference from BL)**  CBT –1.58, C 0.35  **BMI (difference from BL)**  CBT 0.01, C 0.42  **Depression (BDI) (difference from BL)**  CBT –1.86, C 0.96 | **Sampling method**  Permuted block design  **Blinding**  Unclear  **Handling of missing data**  Linear mixed model |
| Shapiro et al  2007  USA  (48) | **Population,** N=66  (86% of eligible, 56% of screened)  Mean age: 39.6±11.4  Mean BMI: 37.72±9.45  92% females  Full BED: 70%  **Inclusion criteria:** Age 18–60, DSM-IV BED-based, sub­threshold BED: ≥two objective binge eating episodes/month, BMI≥27, regular access to an IBM-compatible computer  **Setting:** Outpatients recruited via newspaper advertisements | CBT, n=22  CBT: group treatment for weekly 90-minute sessions, 5–10 participants/group  CD-ROM, n=22  Interactive programme, illustrations, photographs, interactive exercises and video clips  **Duration:** 10 weeks  **Drop out:** 10 weeks: CBT 9 (41%), CD-ROM 7 (32%)  2 months: CBT 13(59%), CD-ROM 14(63%) | Waiting list, n=22  **Duration:** 10 weeks  **Drop out:** 2/22 (9%) | **Abstinence, N (%)**  Post: CD-ROM 2 (13.3%), CBT 1 (7.7%), WL 0  2 months: CD-ROM 1 (12.5%), CBT 2 (22.2%), WL 0  **BES**  Post: CBT 20.92±4.31, CD-ROM 23.4±5.83, WL 23.6±6.14  **Binge days/week**  Post: CBT 2.08±0.67, CD-ROM 2.58±1.56, WL 2.5±1.2  **BMI**  Post: CBT 37.03±10.49, CD-ROM 38.63±9, WL 34.39±6.35 | **Sampling method**  Block design  **Blinding**  Unclear  **Handling of missing data**  ITT analysis |
| ter Huurne et al  2015  Netherlands  (49) | **Population,** N=85  Mean age: 40.2±11.4  BMI >25: 100%  Duration of BED> 11 years: 72%  **Inclusion criteria:** Age ≥18, females, BED according to DSM-IV  **Setting:** Outpatients, announce-ments and advertisements | Web based CBT, n=43  Structured 2 part program, >21 contacts moments and 10 assignments  **Duration:** 15 weeks  **Drop out:** 4 (9%) | Waiting list, n=42  **Duration:** 15 weeks  **Drop out:** 2 (5%) | **EDE-Q Total**  I 2.6±1.3, C 3.2±0.9  **BMI**  I 34.7±6.5, C 34.2±5.4  **QoL, (EQ-5D VAS)**  I 67.9±16, C 62.7±15.5  **Depression (DASS)**  I 8.7±7.5, C 12±8.3 | **Sampling method**  Computer generated block size (2, 4 or 8), stratification 1:1, concealed allocation  **Blinding**  Unclear  **Handling of missing data**  ITT  **Other comments**  We only included data from the BED and not BN and EDNOS population |
| White et al  2013  USA  (50) | **Population,** N=61  (66% of screened)  Mean age: 44±12.5  Mean BMI 35.8±6.8  **Inclusion criteria:** Age 18–65, female, met DSM-IV TR criteria for BED, BMI≥25  **Setting:** Outpatients recruited via advertisements | Bupropion, n=31  300mg/day  **Duration:** 8 weeks  **Drop out:** 4 (13%) | Placebo, n=30  **Duration:** 8 weeks  **Drop out:** 3 (10%) | **Remission (no BE/month)**  I 13 (42%), C 8 (27%)  **EDE restrain**  I 1.4±1, C 1.6±0.8  **EDE-Q Global score**  I 1.8±0.9, C 2±0.9  **OBE, monthly**  I 5±9.4, C 6.3±8  **OBE/week**  I 0.8±1.2, C 1±1.5  **BMI**  I 35.7±6.6, C 35.2±7.4  **Depression (BDI)**  I 8±8.3, C 8.7±7.2  **Adverse events**  No medical events were reported | **Sampling method**  Stratification by obesity grade  And smoking  **Blinding**  Double blind  **Handling of missing data**  ITT analysis, last data carried forward |
| Wilfley et al  2002  (51)  Hilbert et al  2012  USA  (52) | **Population,** N=162  (83% of eligible, 17% of screened)  Mean age: 45.3  Mean BMI: 37.4±5.2  82.7% females  **Inclusion criteria:** Age 18–65, DSM-IV criteria for BED: ≥2 days of BE/wk, ≥6 months, marked distress regarding BE, ≥3 of 5 associated behavioural features, no regular use of inappropriate compensatory behaviour, BMI 27–48  **Setting:** Recruited via advertisements, two sites, outpatient, Eating disorder clinics at Yale University and San Diego State | IPT, n=81  *Both IPT and CBT:*  9 participants/group  20 90-minutes weekly group sessions+3 individual sessions. Manual-based and led by two therapists  *IPT:* focused on problem resolution within 4 social domains: Grief, interpersonal role disputes, role transitions, interpersonal deficits  **Duration:** 23 weeks  **Drop out:**  Post: 1(1.2%)  4 months: 5(6%)  8 months: 6(7.4%)  12 months 10(12%)  4 years: 12(27%) | CBT, n=81  *CBT*: 3 phases focusing on behavioural strategies, cognitive skills and relapse prevention  **Duration:** 23 weeks  **Drop out**  Post: 4 (5%)  4 months: 6(7.4%)  8 months: 10(12%)  12 months: 14(17%)  4 years: 20(44%) | **Binge days/28 days**  Post: CBT 0.6±1.6, IPT 0.9±2  4 months: CBT 2.0±4.6, IPT 1.5±3.9  8 months: CBT 2.1 (5.0), IPT 1.9 (4.5)  12 months: CBT 1.7±4.3, IPT 1.2±2.6  **Remission/Abstinence from binge-eating**  Post: CBT 64 (79%), IPT 59 (72%)  12 months: CBT 48 (59%), IPT 50 (62%)  4 years (N=90): CBT 13/25, IPT 23/30  **Depression (SCL)**  Post: CBT 34.8±7.9, IPT 33.6±8.6  4 months: CBT 34.2±8.3, IPT 34.6±10.6  8 months: CBT 33.3±8.6, IPT 34.4±10.7  12 months: CBT 33.1±8.2, IPT 32.2±10.3  **BMI**  Post: CBT 37.5±5.3, IPT 37.2±5.2  4 months: CBT 37.4±5.3, IPT 36.6±5.3  8 months: CBT 37.5±5.1, IPT 36.4±5.5  12 months: CBT 37.2±5.1, IPT 36.3±5.4  **Depression (SCL)**  Post: CBT 24.8±7.9, IPT 33.6±8.6  4 months: CBT 34.3±8.3, IPT 34.6±10.6  8 months: CBT 33.3±8.6, IPT 34.4±10.7  12 months: CBT 33.1±8.2, IPT 32.2±10.3 | **Sampling method**  NR  **Blinding**  NA for participants Assessors blinded,  **Handling of missing data**  ITT analysis |
| Wilson et al  2010  (53)  Hilbert et al  2015  USA  (54) | **Population,** N=205  (9% of screened)  Mean age: 48  Mean BMI: 36.4  56% females  **Inclusion criteria:** Age ≥18, met DSM-IV criteria for BED, BMI 27–45  **Setting:** Recruited via advertisement and clinical referrals, University outpatient clinics | IPT, n=75  Behavioural weight loss treatment (BWL), n=64  Twenty sessions of IPT or BWL or 10 sessions  **Duration:** 6 months  **Drop out:** Post: IPT: 5/75, BWL: 18/64 | CBT-gsh, n=66  **Duration:** 6 months  **Drop out:** Post: 20/66 | **Remission, %**  Post: IPT 65%, CBT-gsh 60%, BWL 56%  1 year: IPT 57%, CBT-gsh 62%, BWL 44%  2 years: IPT 68%, CBT-gsh 64%, BWL 47%  **No of binge days/28 days**  Post: IPT 3.7±7.2, BWL 4.3±7.9, CBT-gsh 3.8±7.2  1 year: IPT 4.8±7.6, BWL 6.5±8.7, CBT-gsh 4.3±7.8  2 years: IPT 4.3±7.8, BWL 5.8±8.5, CBT-gsh 3.7±7.3  **BMI**  Post: IPT 35.9±5.3, BWL 35.4±5.7, CBT-gsh 36.1±4.4  1 year: IPT 35.9±5.4, BWL 36±6.2, CBT-gsh 35.7±5  2 years: IPT 36.1±5.5, BWL 36.3±6.2, CBT-gsh 35.7±4.9  **EDE-Q Global score**  Post: IPT 1.8±0.9, BWL 2.1±1.0, CBT-gsh 1.7±1.0  1 year: IPT 1.9±1.0, BWL 2.2±1, CBT-gsh 1.7±0.9  2 years: IPT 1.7±1.1, BWL 2±1.2, CBT-gsh 1.7±1  Rapid responder (reduction in BE ≥70 by 4^th^ week of treatment)  BWL 47/64, CBT-gsh 49/66, IPT 49/75 | **Sampling method**  Computer generated sequence, stratification  **Blinding**  Blinded assessors  **Handling of missing data**  ITT analysis |

BDI=Beck Depression Inventory; BDI-II=Beck Depression Inventory II; BED=binge eating disorder; BES=binge eating scale; BMI=body mass index; BST= Brief Strategic Therapy; BWL=behavioural weight loss; CBT= Cognitive behavioural therapy; CBT-C= CBT with cognitive restructuring component focused on body image: CBT-E= CBT with body exposure component; CBT-ED= CBT plus low-Energy-Density diet; CBT-sh= CBT self-help; CBT+WL= CBT+weight loss therapy; CES-D=Center for Epidemiological Studies for Depression; CFT= Self-compassion focused therapy; CGI= Clinical Global Impressions; DASS=Depression Anxiety Stress Scales; DSM= The Diagnostic and Statistical Manual of Mental Disorders edition; EDE= Eating Disorder Examination; EDE-Q= Eating Disorder Examination-Questionnaire; EMM=estimated marginal of means; G-CBT=group-CBT; gsh= guided self-help; HAD=Hospital Anxiety and Depression Scale; HAM-D=Hamilton rating scale for depression; IDS-C= The Inventory of Depressive Symptomatology, Clinician; IDW= ideal body weight; IPT= Interpersonal psychotherapy; ITT=intention To Treat; LDX=lisdexamfetamine; MADRS= Montgomery Åsberg Depression Rating Scale; MB-EAT= Mindfulness-Based Eating Awareness Training; NHP= Nottingham Health Profile; PECB= Psycho-educational/cognitive behaviour; NR=not reported; OBE=objective binge eating; QIDS-SR= Quick Inventory of Depressive Symptomatology-self-report; QoL=Quality of Life; SD=standard deviation; TTD=Three times daily; WL= weight loss therapy

### References:

1. Agras WS, Telch CF, Arnow B, Eldredge K, Wilfley DE, Raeburn SD, et al. Weight loss, cognitive-behavioral, and desipramine treatments in binge eating disorder: An additive design. Behavior therapy. 1994;25:225-38.

2. Alfonsson S, Parling T, Ghaderi A. Group behavioral activation for patients with severe obesity and binge eating disorder: a randomized controlled trial. Behavior modification. 2015;39:270-94.

3. Brownley KA, Von Holle A, Hamer RM, La Via M, Bulik CM. A double-blind, randomized pilot trial of chromium picolinate for binge eating disorder: results of the Binge Eating and Chromium (BEACh) study. Journal of psychosomatic research. 2013;75:36-42.

4. Carrard I, Crépin C, Rouget P, Lam T, Golay A, Linden M. Randomised controlled trial of a guided self-help treatment on the Internet for binge eating disorder. Behav Res Ther. 2011;49:482-91.

5. Carter JC, Fairburn CG. Cognitive-behavioral self-help for binge eating disorder: a controlled effectiveness study. J Consult Clin Psychol. 1998;66:616-23.

6. Cassin SE, von Ranson KM, Heng K, Brar J, Wojtowicz AE. Adapted motivational interviewing for women with binge eating disorder: a randomized controlled trial. Psychology of addictive behaviors : journal of the Society of Psychologists in Addictive Behaviors. 2008;22:417-25.

7. Castelnuovo G, Manzoni GM, Villa V, Cesa GL, Molinari E. Brief Strategic Therapy vs Cognitive Behavioral Therapy for the Inpatient and Telephone-Based Outpatient Treatment of Binge Eating Disorder: The STRATOB Randomized Controlled Clinical Trial. Clinical practice and epidemiology in mental health : CP & EMH. 2011;7:29-37.

8. Claudino AM, de Oliveira IR, Appolinario JC, Cordas TA, Duchesne M, Sichieri R, et al. Double-blind, randomized, placebo-controlled trial of topiramate plus cognitive-behavior therapy in binge-eating disorder. The Journal of clinical psychiatry. 2007;68:1324-32.

9. Corwin RL, Boan J, Peters KF, Ulbrecht JS. Baclofen reduces binge eating in a double-blind, placebo-controlled, crossover study. Behavioural pharmacology. 2012;23:616-25.

10. Dingemans AE, Spinhoven P, van Furth EF. Predictors and mediators of treatment outcome in patients with binge eating disorder. Behaviour research and therapy. 2007;45:2551-62.

11. Golay A, Laurent-Jaccard A, Habicht F, Gachoud JP, Chabloz M, Kammer A, et al. Effect of orlistat in obese patients with binge eating disorder. Obesity research. 2005;13:1701-8.

12. Grilo CM, Masheb RM, White MA, Gueorguieva R, Barnes RD, Walsh BT, et al. Treatment of binge eating disorder in racially and ethnically diverse obese patients in primary care: randomized placebo-controlled clinical trial of self-help and medication. Behaviour research and therapy. 2014;58:1-9.

13. Grilo CM, White MA, Gueorguieva R, Barnes RD, Masheb RM. Self-help for binge eating disorder in primary care: a randomized controlled trial with ethnically and racially diverse obese patients. Behaviour research and therapy. 2013;51:855-61.

14. Grilo CM, White MA. Orlistat with behavioral weight loss for obesity with versus without binge eating disorder: randomized placebo-controlled trial at a community mental health center serving educationally and economically disadvantaged Latino/as. Behaviour research and therapy. 2013;51:167-75.

15. Grilo CM, Masheb RM, Wilson GT, Gueorguieva R, White MA. Cognitive-behavioral therapy, behavioral weight loss, and sequential treatment for obese patients with binge-eating disorder: a randomized controlled trial. J Consult Clin Psychol. 2011;79:675-85.

16. Grilo CM, Masheb RM. A randomized controlled comparison of guided self-help cognitive behavioral therapy and behavioral weight loss for binge eating disorder. Behaviour research and therapy. 2005;43:1509-25.

17. Masheb RM, Grilo CM. Rapid response predicts treatment outcomes in binge eating disorder: implications for stepped care. J Consult Clin Psychol. 2007;75:639-44.

18. Masheb RM, Grilo CM. Prognostic significance of two sub-categorization methods for the treatment of binge eating disorder: negative affect and overvaluation predict, but do not moderate, specific outcomes. Behaviour research and therapy. 2008;46:428-37.

19. Grilo CM, Masheb RM, Salant SL. Cognitive behavioral therapy guided self-help and orlistat for the treatment of binge eating disorder: a randomized, double-blind, placebo-controlled trial. Biological psychiatry. 2005;57:1193-201.

20. Grilo CM, Masheb RM, Wilson GT. Efficacy of cognitive behavioral therapy and fluoxetine for the treatment of binge eating disorder: a randomized double-blind placebo-controlled comparison. Biological psychiatry. 2005;57:301-9.

21. Grilo CM, Masheb RM, Wilson GT. Rapid response to treatment for binge eating disorder. J Consult Clin Psychol. 2006;74:602-13.

22. Grilo CM, Crosby RD, Wilson GT, Masheb RM. 12-month follow-up of fluoxetine and cognitive behavioral therapy for binge eating disorder. J Consult Clin Psychol. 2012;80:1108-13.

23. Guerdjikova AI, McElroy SL, Winstanley EL, Nelson EB, Mori N, McCoy J, et al. Duloxetine in the treatment of binge eating disorder with depressive disorders: a placebo-controlled trial. The International journal of eating disorders. 2012;45:281-9.

24. Guerdjikova AI, McElroy SL, Welge JA, Nelson E, Keck PE, Hudson JI. Lamotrigine in the treatment of binge-eating disorder with obesity: a randomized, placebo-controlled monotherapy trial. International clinical psychopharmacology. 2009;24:150-8.

25. Guerdjikova AI, McElroy SL, Kotwal R, Welge JA, Nelson E, Lake K, et al. High-dose escitalopram in the treatment of binge-eating disorder with obesity: a placebo-controlled monotherapy trial. Human psychopharmacology. 2008;23:1-11.

26. Hilbert A, Tuschen-Caffier B. Body image interventions in cognitive-behavioural therapy of binge-eating disorder: a component analysis. Behaviour research and therapy. 2004;42:1325-39.

27. Hudson JI, McElroy SL, Raymond NC, Crow S, Keck PE, Jr., Carter WP, et al. Fluvoxamine in the treatment of binge-eating disorder: a multicenter placebo-controlled, double-blind trial. The American journal of psychiatry. 1998;155:1756-62.

28. Kelly AC, Carter JC. Self-compassion training for binge eating disorder: A pilot randomized controlled trial. Psychol Psychother. 2014;0.

29. Kristeller J, Wolever RQ, Sheets V. Mindfulness-Based Eating Awareness Training (MB-EAT) for binge eating: A randomized clinical trial. Mindfulness. 2014;5:282-97.

30. Laederach-Hofmann K, Graf C, Horber F, Lippuner K, Lederer S, Michel R, et al. Imipramine and diet counseling with psychological support in the treatment of obese binge eaters: a randomized, placebo-controlled double-blind study. International journal of eating disorders. 1999;26:231-44.

31. Leombruni P, Piero A, Lavagnino L, Brustolin A, Campisi S, Fassino S. A randomized, double-blind trial comparing sertraline and fluoxetine 6-month treatment in obese patients with Binge Eating Disorder. Progress in neuro-psychopharmacology & biological psychiatry. 2008;32:1599-605.

32. Masheb RM, Grilo CM, Rolls BJ. A randomized controlled trial for obesity and binge eating disorder: low-energy-density dietary counseling and cognitive-behavioral therapy. Behaviour research and therapy. 2011;49:821-9.

33. Masson PC, von Ranson KM, Wallace LM, Safer DL. A randomized wait-list controlled pilot study of dialectical behaviour therapy guided self-help for binge eating disorder. Behaviour research and therapy. 2013;51:723-8.

34. McElroy SL, Hudson J, Ferreira-Cornwell MC, Radewonuk J, Whitaker T, Gasior M. Lisdexamfetamine Dimesylate for Adults with Moderate to Severe Binge Eating Disorder: Results of Two Pivotal Phase 3 Randomized Controlled Trials. Neuropsychopharmacology. 2015;0.

35. McElroy SL, Guerdjikova AI, Mori N, Blom TJ, Williams S, Casuto LS, et al. Armodafinil in binge eating disorder: a randomized, placebo-controlled trial. International clinical psychopharmacology. 2015;30(4):209-15.

36. McElroy SL, Hudson JI, Mitchell JE, Wilfley D, Ferreira-Cornwell MC, Gao J, et al. Efficacy and safety of lisdexamfetamine for treatment of adults with moderate to severe binge-eating disorder: a randomized clinical trial. JAMA psychiatry. 2015;72:235-46.

37. McElroy SL, Hudson JI, Capece JA, Beyers K, Fisher AC, Rosenthal NR. Topiramate for the treatment of binge eating disorder associated with obesity: a placebo-controlled study. Biological psychiatry. 2007;61:1039-48.

38. McElroy SL, Hudson JI, Malhotra S, Welge JA, Nelson EB, Keck PE, Jr. Citalopram in the treatment of binge-eating disorder: a placebo-controlled trial. The Journal of clinical psychiatry. 2003;64:807-13.

39. McElroy SL, Casuto LS, Nelson EB, Lake KA, Soutullo CA, Keck PE, Jr., et al. Placebo-controlled trial of sertraline in the treatment of binge eating disorder. The American journal of psychiatry. 2000;157:1004-6.

40. McIntosh VVW, Jordan J, Carter JD, Frampton CMA, McKenzie JM, Latner JD, et al. Psychotherapy for transdiagnostic binge eating: A randomized controlled trial of cognitive-behavioural therapy, appetite-focused cognitive-behavioural therapy, and schema therapy. Psychiatry research. 2016;240:412-20.

41. Munsch S, Biedert E, Meyer A, Michael T, Schlup B, Tuch A, et al. A randomized comparison of cognitive behavioral therapy and behavioral weight loss treatment for overweight individuals with binge eating disorder. The International journal of eating disorders. 2007;40:102-13.

42. Munsch S, Meyer AH, Biedert E. Efficacy and predictors of long-term treatment success for Cognitive-Behavioral Treatment and Behavioral Weight-Loss-Treatment in overweight individuals with binge eating disorder. Behaviour research and therapy. 2012;50:775-85.

43. Pearlstein T, Spurell E, Hohlstein LA, Gurney V, Read J, Fuchs C, et al. A double-blind, placebo-controlled trial of fluvoxamine in binge eating disorder: a high placebo response. Archives of women's mental health. 2003;6:147-51.

44. Peterson CB, Mitchell JE, Crow SJ, Crosby RD, Wonderlich SA. The efficacy of self-help group treatment and therapist-led group treatment for binge eating disorder. The American journal of psychiatry. 2009;166:1347-54.

45. Ricca V, Castellini G, Mannucci E, Lo Sauro C, Ravaldi C, Rotella CM, et al. Comparison of individual and group cognitive behavioral therapy for binge eating disorder. A randomized, three-year follow-up study. Appetite. 2010;55:656-65.

46. Schlup B, Munsch S, Meyer AH, Margraf J, Wilhelm FH. The efficacy of a short version of a cognitive-behavioral treatment followed by booster sessions for binge eating disorder. Behaviour research and therapy. 2009;47:628-35.

47. Fischer S, Meyer AH, Dremmel D, Schlup B, Munsch S. Short-term cognitive-behavioral therapy for binge eating disorder: long-term efficacy and predictors of long-term treatment success. Behaviour research and therapy. 2014;58:36-42.

48. Shapiro JR, Reba-Harrelson L, Dymek-Valentine M, Woolson SL, Hamer RM, Bulik CM. Feasibility and acceptability of CD-ROM-based cognitive-behavioural treatment for binge-eating disorder. European eating disorders review : the journal of the Eating Disorders Association. 2007;15:175-84.

49. ter Huurne ED, de Haan HA, Postel MG, van der Palen J, VanDerNagel JE, DeJong CA. Web-Based Cognitive Behavioral Therapy for Female Patients With Eating Disorders: Randomized Controlled Trial. Journal of medical Internet research. 2015;17(6):e152.

50. White MA, Grilo CM. Bupropion for overweight women with binge-eating disorder: a randomized, double-blind, placebo-controlled trial. The Journal of clinical psychiatry. 2013;74:400-6.

51. Wilfley DE, Welch RR, Stein RI, Spurrell EB, Cohen LR, Saelens BE, et al. A randomized comparison of group cognitive-behavioral therapy and group interpersonal psychotherapy for the treatment of overweight individuals with binge-eating disorder. Archives of general psychiatry. 2002;59:713-21.

52. Hilbert A, Bishop ME, Stein RI, Tanofsky-Kraff M, Swenson AK, Welch RR, et al. Long-term efficacy of psychological treatments for binge eating disorder. The British journal of psychiatry : the journal of mental science. 2012;200:232-7.

53. Wilson GT, Wilfley DE, Agras WS, Bryson SW. Psychological treatments of binge eating disorder. Archives of general psychiatry. 2010;67(1):94-101.

54. Hilbert A, Hildebrandt T, Agras WS, Wilfley DE, Wilson GT. Rapid response in psychological treatments for binge eating disorder. J Consult Clin Psychol. 2015;83(3):649-54.
